# Surrogate- and invariance-boosted contrastive learning for data-scarce applications in science

**DOI:** 10.1038/s41467-022-31915-y

**Published:** 2022-07-21

**Authors:** Charlotte Loh, Thomas Christensen, Rumen Dangovski, Samuel Kim, Marin Soljačić

**Affiliations:** 1 Department of Electrical Engineering and Computer Science, Massachusetts Institute of Technology, Cambridge, MA USA; 2 Department of Physics, Massachusetts Institute of Technology, Cambridge, MA USA

**Keywords:** Computer science, Optics and photonics, Theory and computation, Physics

## Abstract

Deep learning techniques have been increasingly applied to the natural sciences, e.g., for property prediction and optimization or material discovery. A fundamental ingredient of such approaches is the vast quantity of labeled data needed to train the model. This poses severe challenges in data-scarce settings where obtaining labels requires substantial computational or labor resources. Noting that problems in natural sciences often benefit from easily obtainable auxiliary information sources, we introduce surrogate- and invariance-boosted contrastive learning (SIB-CL), a deep learning framework which incorporates three inexpensive and easily obtainable auxiliary information sources to overcome data scarcity. Specifically, these are: abundant unlabeled data, prior knowledge of symmetries or invariances, and surrogate data obtained at near-zero cost. We demonstrate SIB-CL’s effectiveness and generality on various scientific problems, e.g., predicting the density-of-states of 2D photonic crystals and solving the 3D time-independent Schrödinger equation. SIB-CL consistently results in orders of magnitude reduction in the number of labels needed to achieve the same network accuracies.

## Abstract (web summary)

Deep learning techniques usually require a large quantity of training data and may be challenging for scarce datasets. The authors propose a framework that involves contrastive and transfer learning and reduces data requirements for training while keeping the prediction accuracy.

## Introduction

In recent years, there has been increasing interest and rapid advances in applying data-driven approaches, in particular, deep learning via neural networks, to problems in the natural sciences^1–4^. Unlike traditional physics-informed approaches, deep learning relies on extensive amounts of data to quantitatively discover hidden patterns and correlations to perform tasks such as predictive modeling^4,5^, property optimization^6,7^, and knowledge discovery^8,9^. Its success is thus largely contingent on the amount of data available and a lack of sufficient data can severely impair model accuracy. Historically, deep learning applications have overcome this by brute-force, e.g., by assembling vast curated data sets by crowd-sourced annotation or from historical records. Prominent examples include ImageNet^10^ and CIFAR^11^ in computer vision (CV) and WordNet^12^ in natural language processing (NLP) applications. The majority of problems in the natural sciences, however, are far less amenable to this brute-force approach, partly reflecting a comparative lack of historical data, and partly the comparatively high resource-cost (e.g., time or labor) of synthesizing new experimental or computational data.

A popular approach to alleviate the reliance on labeled data is transfer learning (TL)^13–17^, which refers to the strategy of fine-tuning a neural network which has been pre-trained on a large labeled source dataset for a target task. TL has been explored and proven effective in various works within the natural sciences ^18–21^; however, most works often make use of source data from a different problem^18,19^ or domain^20,21^ thus limiting the efficacy of TL due to the dissimilarity between the source and target problems^22,23^. In this work, we overcome this limitation by using a prominent feature unique to problems in the natural sciences—that they often benefit from exact and approximate analytical techniques or general insights requiring minimal or no computational cost. While this concept was previously explored by Zhang and Ling^24^, their method uses kernel ridge regression (KRR) and thus cannot take advantage of inductive biases commonly used in deep learning without specialized kernels.

More recently, an increasingly popular technique is that of self-supervised learning (SSL)^25,26^, which primarily differs from TL in that the pre-training stage uses unlabeled rather than labeled data. Specifically, pretext tasks like image rotation prediction^27^ and jigsaw puzzle solving^28^ are invented for the data to provide its own supervision. In particular, contrastive SSL^26^ (or contrastive learning) is an increasingly popular technique where the pretext task is constructed as contrasting between two variations of a sample and other samples, where variations are derived using image transformations. The goal is for the pre-trained model to output embeddings where similar (differing) instances are closer (further) in the embedding metric space. In this work, we leverage contrastive learning to invoke symmetries in the problem.

Exploiting physical insights and symmetries has been a highly effective strategy in scientific machine learning. For example, in molecular sciences, symmetry knowledge is often invoked via hand-crafted features^29^ or using deep tensor neural networks^30–33^ with components analytically formulated to respect physical laws and create chemistry-related inductive biases. In these works, invariance is achieved either through the parameterization of the inputs^30,31^ or due to intrinsic symmetry preservation of the architecture itself^32,33^. These approaches are, however, highly domain-specific since the architecture is either hand-crafted or analytically formulated specifically for atomistic systems. Equivariant neural networks^34–37^ provides a domain-agnostic alternative to exploit symmetries; they have been generalized to spherical images^38^, volumetric data^37^, and has been effectively applied to the natural sciences^39,40^ as well. However, the design of such architectures still involves deep technical expertize to mathematically construct the symmetry-preserving specialized kernels. In contrast, contrastive learning provides a domain- and model-agnostic approach to exploit symmetries, where a black-box approach is used to embed physical knowledge instead of through the network architecture or input parameterization, bearing some similarities to conventional data augmentation^41–44^ strategies. While some applications have been explored in various works^45–48^, most are confined within graph architectures; applications to the natural sciences have been scarce, partly owing to the intricacy of designing transformation strategies^49^ suitable for scientific problems.

**Fig. 1:**
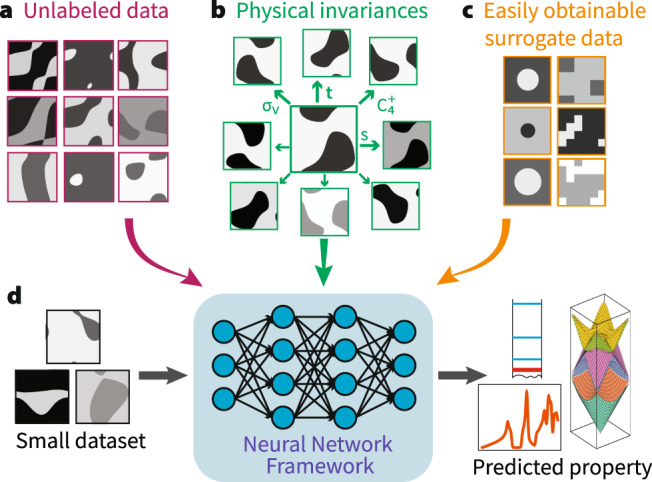
Overcoming data scarcity with SIB-CL. We propose to overcome data scarcity by leveraging **a** an abundance of unlabeled data, **b** prior knowledge of the underlying physics (e.g., symmetries and invariances of the data), and **c** knowledge from a possibly-approximate surrogate data which is faster and cheaper to generate (e.g., coarse-grained computations or special-case analytical solutions). **d** SIB-CL incorporates these auxiliary information into a single framework to accelerate training in data-scarce settings. Examples show unit cells of square 2D photonic crystals (see also Fig. 3).

Here, we introduce Surrogate- and Invariance- boosted Contrastive Learning (SIB-CL), a deep learning framework based on the unique disposition of problems in natural sciences, where auxiliary information sources are often accessible a priori or can be obtained by inexpensive means (see Fig. 1). Specifically, these are: (1) abundant unlabeled data; (2) prior knowledge in the form of invariances of the physical problem, which can be governed by geometric symmetries of the inputs or general non-symmetry related invariances of the problem; (3) a surrogate dataset on a similar problem that is cheaper to generate, e.g., by invoking simplifications or approximations to the labeling process. SIB-CL uses popular deep learning techniques of TL and SSL as effective and broadly-applicable strategies to incorporate these auxiliary information sources, enabling effective and high-quality network training despite data scarcity. SIB-CL is applicable to domains where a related and simplified surrogate dataset can be created, common to scientific disciplines where approximate or analytical approaches^50–52^ are prevalent. Examples of such domains include the use of classical inter-atomic force fields in molecular dynamics^51^ and the hierarchy of approximations in density-functional theory (DFT) captured by multiple distinct rungs of Jacob’s ladder^52^. Here, SIB-CL’s effectiveness will be demonstrated in various problems in the natural sciences, in particular, on two systems in the fields of photonics and quantum physics calculations.

## Results

### Surrogate- and invariance-boosted contrastive learning (SIB-CL)

We seek to train a neural network to predict desired properties (or labels) **y** from input **x** using minimal training data. More precisely, for a target problem ${D}_{{{{{{{{\rm{t}}}}}}}}}={\{{{{{{{{{\bf{x}}}}}}}}}_{i},{{{{{{{{\bf{y}}}}}}}}}_{i}\}}_{i = 1}^{{N}_{{{{{{{{\rm{t}}}}}}}}}}$ consisting of *N*_t_ input-label pairs, we focus on problem spaces where *N*_t_ is too small to successfully train the associated network. To overcome this, we introduce two auxiliary data sets: (1) a set of zero-cost unlabeled inputs ${D}_{{{{{{{{\rm{u}}}}}}}}}={\{{{{{{{{{\bf{x}}}}}}}}}_{i}\}}_{i = 1}^{{N}_{{{{{{{{\rm{u}}}}}}}}}}$ and (2) a surrogate data set ${D}_{{{{{{{{\rm{s}}}}}}}}}={\{{\tilde{{{{{{{{\bf{x}}}}}}}}}}_{i},{\tilde{{{{{{{{\bf{y}}}}}}}}}}_{i}\}}_{i = 1}^{{N}_{{{{{{{{\rm{s}}}}}}}}}}$ consisting of inexpensively computed labels ${\tilde{{{{{{{{\bf{y}}}}}}}}}}_{i}$ (e.g., from approximation or semi-analytical models) with associated input ${\tilde{{{{{{{{\bf{x}}}}}}}}}}_{i}$ (possibly, but not necessarily, a “simple” subset of all inputs). The quantity of each of these auxiliary data sets are assumed to far exceed the target problem, i.e., {*N*_u_, *N*_s_} ≫ *N*_t_ (and, typically, *N*_u_ > *N*_s_).

**Fig. 2:**
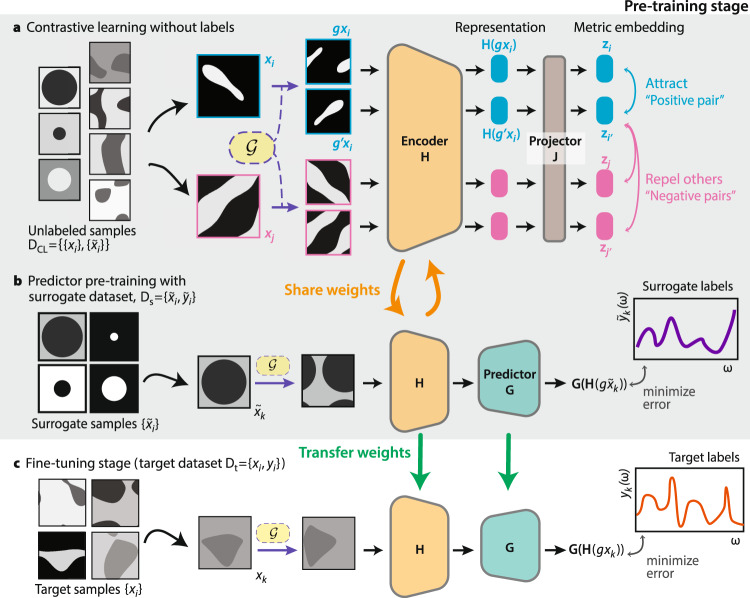
Surrogate- and invariance-boosted contrastive learning (SIB-CL) framework. Network training proceeds via a pre-training stage (**a**, **b**) followed by a fine-tuning stage (**c**). The pre-training stage alternates a contrastive learning step **a** using unlabeled data *D*_CL_ with a supervised learning step **b** using surrogate data *D*_s_. Contrastive learning encourages representations that respect the underlying invariances of the problem and supervised learning on the surrogate dataset attunes both representations and a predictor network to the desired prediction task. **c** After 100–400 epochs of pre-training, the encoder, and predictor weights are copied and subsequently fine-tuned by supervised learning on the target dataset *D*_t_.

On the basis of these auxiliary datasets, we introduce our framework—Surrogate and Invariance-Boosted Constrastive Learning (SIB-CL)—that significantly reduces the data requirements on *D*_t_ (Fig. 2). SIB-CL achieves this by splitting the training process into two stages: a first, interleaved two-step pre-training stage using the auxiliary data sets *D*_u_ and *D*_s_ (Fig. 2a, b), followed by a fine-tuning stage using the target data set *D*_t_ (Fig. 2c).

In the first step of the pre-training stage (Fig. 2a), we exploit contrastive learning to learn invariances in the problem space using unlabeled inputs aggregated from the target and surrogate data sets ${D}_{{{{{{{{\rm{CL}}}}}}}}}={\{{{{{{{{{\bf{x}}}}}}}}}_{i}\}}_{i = 1}^{{N}_{{{{{{{{\rm{u}}}}}}}}}}\cup {\{{\tilde{{{{{{{{\bf{x}}}}}}}}}}_{i}\}}_{i = 1}^{{N}_{{{{{{{{\rm{s}}}}}}}}}}$. We complement *D*_CL_ by a set of known, physics-informed invariance relations {*g*} (which we formally associate with elements of a group ${{{{{{{\mathcal{G}}}}}}}}$) which map input–label pairs (**x**_*i*_, **y**_*i*_) to (*g***x**_*i*_, **y**_*i*_), i.e., to new input with identical labels. We base this step on the SimCLR technique^53^, though we also explore using the BYOL technique^54^ later (see “Discussion” and SI section S1). Specifically, for each input **x**_*i*_ in *D*_CL_ (sampled in batches of size *B*), two derived variations *g***x**_*i*_ and $g^{\prime} {{{{{{{{\bf{x}}}}}}}}}_{i}$ are created by sampling two concrete mappings *g* and $g^{\prime}$ from the group of invariance relations ${{{{{{{\mathcal{G}}}}}}}}$ (see Methods). The resultant 2*B* inputs are then fed into encoder and then projector networks, **H** and **J** respectively, producing metric embeddings ${{{{{{{{\bf{z}}}}}}}}}_{{i}^{({\prime} )}}={{{{{{{\bf{J}}}}}}}}({{{{{{{\bf{H}}}}}}}}({g}^{({\prime} )}{{{{{{{{\bf{x}}}}}}}}}_{i}))$. A positive pair $\{{{{{{{{{\bf{z}}}}}}}}}_{i},{{{{{{{{\bf{z}}}}}}}}}_{i^{\prime} }\}$ is the pair of metric embeddings derived from the two variations of **x**_*i*_, i.e., *g***x**_*i*_ and $g^{\prime} {{{{{{{{\bf{x}}}}}}}}}_{i}$; all other pairings in the batch are considered negative. At each training step, the weights of **H** and **J** are simultaneously updated according to a contrastive loss function defined by the normalized temperature-scaled cross entropy (NT-Xent) loss^53^:

1 $${{{{{{{{\mathcal{L}}}}}}}}}_{ii^{\prime} }=-\log \frac{\exp ({s}_{ii^{\prime} }/\tau )}{{\sum }_{j=1}^{2B}[i\ne j]\exp ({s}_{ij}/\tau )},$$

where ${s}_{ii^{\prime} }={\hat{{{{{{{{\bf{z}}}}}}}}}}_{i}\cdot {\hat{{{{{{{{\bf{z}}}}}}}}}}_{i^{\prime} }$ (and ${\hat{{{{{{{{\bf{z}}}}}}}}}}_{i}={{{{{{{{\bf{z}}}}}}}}}_{i}/\left\Vert {{{{{{{{\bf{z}}}}}}}}}_{i}\right\Vert$) denotes the cosine similarity between two normalized metric embeddings ${\hat{{{{{{{{\bf{z}}}}}}}}}}_{i}$ and ${\hat{{{{{{{{\bf{z}}}}}}}}}}_{i^{\prime} }$, [*i* ≠ *j*] uses the Iverson bracket notation, i.e., evaluating to 1 if *i* ≠ *j* and 0 otherwise, and *τ* is a temperature hyperparameter (fixed at 0.1 here). The total loss is taken as the sum across all positive pairs in the batch. In our batch sampling of *D*_CL_, we sample one-third of each batch from *D*_s_ and two-thirds from *D*_u_. Conceptually, the NT-Xent loss acts to minimize the distance between embeddings of positive pairs (numerator of Eq. (1)) while maximizing the distances between embeddings of negative pairs in the batch (denominator of Eq. (1)) Consequently, we obtain representations **H**(**x**_*i*_) that respect the underlying invariances of the problem.

Each epoch of contrastive learning (i.e., each full sampling of *D*_CL_) is followed by a supervised learning step—the second step of the pre-training stage (Fig. 2b)—on the surrogate dataset *D*_s_, with each input from *D*_s_ subjected to a random invariance mapping. This supervised step shares the encoder network **H** with the contrastive step but additionally features a predictor network **G**, both updated via a task-dependent supervised training loss function (which will be separately detailed later). This step pre-conditions the weights of **G** and further tunes the weights of **H** to suit the target task.

The pre-training stage is performed for 100–400 epochs and is followed by the fine-tuning stage (Fig. 2c). This final stage uses *D*_t_ to fine-tune the networks **H** and **G** to the actual problem task—crucially, with significantly reduced data requirements on *D*_t_. Each input from *D*_t_ is also subjected to a random invariance mapping; the associated supervised training loss function is again problem-dependent and may even differ from that used in the pre-training stage.

In the following sections, we evaluate the effectiveness of SIB-CL on two problems: predicting the density-of-states (DOS) of two-dimensional (2D) photonic crystals (PhCs) and predicting the ground state energy of the three-dimensional (3D) non-interacting Schrödinger equation (see SI for additional experiments, including predictions of 2D PhC band structures). To investigate the effectiveness of various auxiliary information sources used in SIB-CL, we benchmark our results against the following baselines:

1. Direct supervised learning (SL): randomly initialized networks **H** and **G** are trained using supervised learning on only the target dataset *D*_t_. This reflects the performance of conventional supervised learning, i.e., without exploiting any auxiliary data sources.
2. Conventional transfer learning (TL): networks **H** and **G** are first pre-trained using supervised learning on the surrogate dataset *D*_s_ and then subsequently fine-tuned on *D*_t_. This reflects the performance of including surrogate information via conventional transfer learning on a desirable well-aligned transfer task .
3. Supervised Learning with invariances (SL-I): each input is subjected to a transformation randomly sampled from {*g*} each time before it is fed into network **H** and trained as per SL. This reflects the performance boost when incorporating invariance information via a standard data augmentation approach.

Finally, to critically evaluate SIB-CL’s effectiveness in incorporating these auxiliary information sources, we contrast SIB-CL with the combination of items 2 and 3 above, i.e., transfer learning with invariances (TL-I). Notably, both SIB-CL and TL-I leverage an equal level of auxiliary information—both invariances and a simplified surrogate dataset—and vary only in their learning algorithm.

### Data generation for 2D photonic crystals

Photonic crystals (PhC) are wavelength-scale periodically-structured materials, whose dielectric profiles are engineered to create exotic optical properties not found in bulk materials, such as photonic band gaps and negative refractive indices, with wide-ranging applications in photonics^55,56^. Prominently among these applications is PhC’s ability to engineer the strength of light-matter interactions^56^—or, equivalently, the density of states (DOS) of photonic modes. The DOS captures the number of modes accessible in a spectral range, i.e., the number of modes accessible to an spectrally narrow emitter, directly affecting e.g., spontaneous and stimulated emission rates. However, computing the DOS is expensive: it requires dense integration across the full Brillouin zone (BZ) of the PhC and summation over bands. Below, we demonstrate that SIB-CL enables effective training of a neural network for prediction of the DOS in 2D PhCs, using only hundreds to thousands of target samples, dramatically reducing DOS-computation costs. Such neural networks could help to accelerate the design of PhC features, either directly via backpropagation^57^ or by offering a cheap evaluation for multiple invocations of the model, replacing conventional design techniques like topology optimization^58^ and inverse design^59^.

**Fig. 3:**
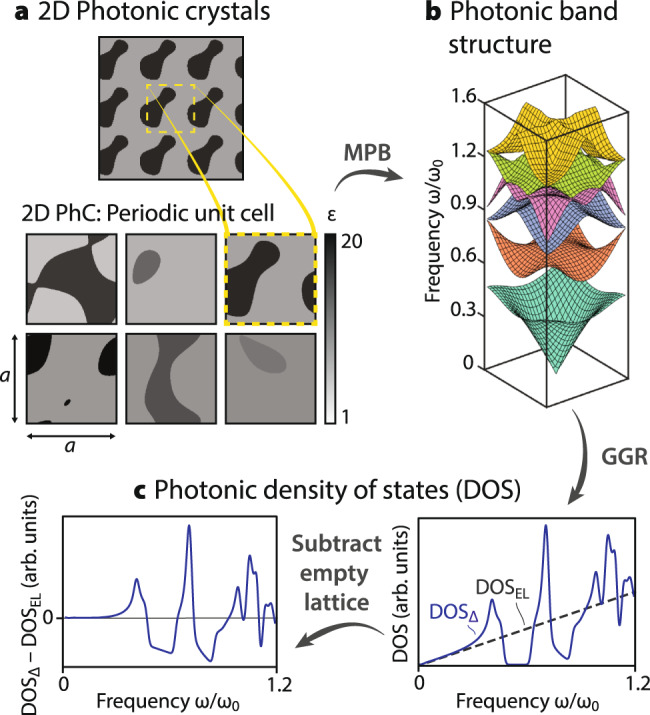
2D photonic crystals dataset generation. **a** We generated 20000 square 2D PhC unit cells using a level set of Fourier sums, giving two-tone permittivity profiles *ε*(**r**) ∈ {*ε*_1_, *ε*_2_} with *ε*_*i*_ ∈ [1, 20]. 7000 of these unit cells were randomly selected; **b** their TM photonic band structures were computed via MPB, and **c** their corresponding density-of-states (DOS) were computed. The DOS spectrums were then smoothed, standardized, and the “empty-lattice” DOS were subtracted from them to derive the labels of the dataset.

PhCs are characterized by a periodically varying permittivity, *ε*(**r**), whose tiling makes up the PhC’s structure. For simplicity, we consider 2D square lattices of periodicity *a* with a “two-tone” permittivity profile, i.e., *ε* ∈ {*ε*_1_, *ε*_2_}, with *ε*_*i*_ ∈ [1, 20]. We assume lossless isotropic materials so that *ε*(**r**) and the resultant eigenfrequencies are real. We use a level-set of a Fourier sum function (see Methods for details) to parameterize *ε*(**r**), discretized to result in a 32 × 32 pixel image, which form the input to the neural network. Special care was taken in the sampling algorithm to create diverse unit cells with features of varying sizes, with examples depicted in Fig. 3a.

We define the DOS of 2D PhCs by^60^

2 $${{{{{{{\rm{DOS}}}}}}}}(\omega )=\frac{A}{{(2\pi )}^{2}}\mathop{\sum}\limits_{n}{\int}_{{{{{{{{\rm{BZ}}}}}}}}}\delta (\omega -{\omega }_{n{{{{{{{\bf{k}}}}}}}}})\,{{{{{{{{\rm{d}}}}}}}}}^{2}{{{{{{{\bf{k}}}}}}}},$$

with *ω* denoting the considered frequency, *ω*_*n***k**_ the PhC band structure, *n* the band index, **k** the momentum in the BZ and *A* = *a*^2^ the unit cell area. In practice, we evaluate Eq. (2) using the generalized Gilat–Raubenheimer (GGR) method^61^—which incorporates the band group velocity extrapolatively to accelerate convergence—in an implementation adapted from ref. ^62^. The band structure and associated group velocities are evaluated using the MIT Photonic Bands (MPB) software^63^ for the transverse magnetic (TM) polarized bands (Fig. 3b, also see Methods).

We define labels for our network using the computed DOS values (Fig. 3c) subjected to three simple post-processing steps (see Methods): (i) spectral smoothing using a narrow Gaussian kernel *S*_Δ_, (ii) shifting by the DOS of the “empty-lattice” (i.e., uniform lattice of index *n*_avg_), ${{{{{{{{\rm{DOS}}}}}}}}}_{{{{{{{{\rm{EL}}}}}}}}}(\omega )=\omega {a}^{2}{n}_{{{{{{{{\rm{avg}}}}}}}}}^{2}/2\pi {c}^{2}$, and (iii) rescaling *both* DOS- and the frequency-values by the natural frequency *ω*_0_ = 2π*c*/*an*_avg_. More explicitly, we define the network-provided DOS labels as

3 $${{{{{{{\bf{y}}}}}}}}\,\triangleq\, {\omega }_{0}[({S}_{{{\Delta }}}* {{{{{{{\rm{DOS}}}}}}}})-{{{{{{{{\rm{DOS}}}}}}}}}_{{{{{{{{\rm{EL}}}}}}}}}](\omega /{\omega }_{0}),$$

and sample over the normalized spectral range 0 ≤ *ω*/*ω*_0_ ≤ 0.96. Step (i) accounts for the finite spectral width of physical measurements and regularizes logarithmic singularities associated with van Hove points; step (ii) counteracts the linear increase in average DOS that otherwise leads to a bias at higher frequencies, emphasizing instead the local spectral features of the DOS; and step (iii) ensures comparable input- and output-ranges across distinct unit cells, regardless of the cell’s average index.

In our experiments, we use 20,000 unlabeled unit cells for contrastive learning, select a target dataset of varying sizes *N*_t_ ∈ [50, 3000] for fine-tuning, and evaluate the prediction accuracy on a fixed test set containing 2000 samples.

For the surrogate dataset of inexpensive data, *D*_s_, we created a simple dataset of 10000 PhCs with centered circular inclusions of varying radii *r* ∈ (0, *a*/2] and inclusion and cladding permittivities sampled uniformly in *ε*_*i*_ ∈ [1, 20]. This simple class of 2D PhCs is amenable to semi-analytical treatments, e.g., Korringa-Kohn–Rostoker or multiple scattering approaches^64–67^, that enable evaluation of the DOS at full precision with minimal computational cost. Motivated by this, we populate the surrogate dataset *D*_s_ with such circular inclusions and their associated (exact) DOS-labels (here, we computed their labels using MPB directly since we are motivated mainly by proof-of-principle rather than concrete applications—and we had access to a preponderance of computational resources provided by MIT Supercloud).

### Invariances of the PhC DOS

The considered PhCs possess no spatial symmetries beyond periodicity. Despite this, as an intrinsic, global quantity (or, equivalently, a **k**-integrated quantity) the DOS is setting-independent and invariant under all size-preserving transformations, that is, under all elements of the Euclidean group *E*(2). For simplicity’s sake, we restrict our focus to the elements of *E*(2) that are compatible with a pixelized unit cell (i.e., that map pixel coordinates to pixel coordinates). This subset is the direct product of the 4mm (*C*_4*v*_) point group ${{{{{{{{\mathcal{G}}}}}}}}}_{0}$ of the point lattice spanned by $a\hat{{{{{{{{\bf{x}}}}}}}}}$ and $a\hat{{{{{{{{\bf{y}}}}}}}}}$ and the group ${{{{{{{{\mathcal{G}}}}}}}}}_{{{{{{{{\bf{t}}}}}}}}}$ of pixel-discrete translations. In more detail:

1. Point group symmetry (${{{{{{{{\mathcal{G}}}}}}}}}_{0}$): 4 mm includes the identity operation (1), 2- and 4-fold rotations (*C*_2_ and ${C}_{4}^{\pm }$), and horizontal, vertical, and diagonal mirrors (*σ*_*h*_, *σ*_*v*_, and ${\sigma }_{d}^{({\prime} )}$), i.e., ${{{{{{{{\mathcal{G}}}}}}}}}_{0}=\{1,{C}_{2},{C}_{4}^{-},{C}_{4}^{+},{\sigma }_{h},{\sigma }_{v},{\sigma }_{d},{\sigma }_{d}^{\prime}\}$. Formally, this is the PhCs’ holosymmetric point group.
2. Translation symmetry (${{{{{{{{\mathcal{G}}}}}}}}}_{{{{{{{{\bf{t}}}}}}}}}$): While the DOS is invariant under all continuous translations **t**, the pixelized unit cells are compatible only with pixel-discrete translations; i.e., we consider the (factor) group ${{{{{{{{\mathcal{G}}}}}}}}}_{{{{{{{{\bf{t}}}}}}}}}={\{i{N}^{-1}a\hat{{{{{{{{\bf{x}}}}}}}}}+j{N}^{-1}a\hat{{{{{{{{\bf{y}}}}}}}}}\}}_{i,j = 0}^{N-1}$ with *N* = 32.

Additionally, the structure of the Maxwell equations endows the DOS with two non-Euclidean “scaling” invariances^55^:

3. Refractive scaling (${{{{{{{{\mathcal{G}}}}}}}}}_{{{{{{{{\rm{s}}}}}}}}}$): The set of (positive) amplitude-scaling transformations of the refractive index *g*(*s*)*n*(**r**) = *sn*(**r**) define a group ${{{{{{{{\mathcal{G}}}}}}}}}_{{{{{{{{\rm{s}}}}}}}}}=\{g(s)\,|\,s\in {{\mathbb{R}}}_{+}\}$ and map the PhC eigenspectrum from *ω*_*n***k**_ to *s*^−1^*ω*_*n***k**_. Equivalently, *g*(*s*) maps DOS(*ω*) to *s*DOS(*sω*) and thus leaves the **y**-labels of Eq. (3) invariant under the *ω*_0_-normalization.
4. Size scaling (${{{{{{{{\mathcal{G}}}}}}}}}_{{{{{{{{\rm{s}}}}}}}}}^{\prime}$): Analogously, the size-scaling transformations $g^{\prime} (s){{{{{{{\bf{r}}}}}}}}=s{{{{{{{\bf{r}}}}}}}}$ define a group ${{{{{{{{\mathcal{G}}}}}}}}}_{{{{{{{{\rm{s}}}}}}}}}^{\prime}=\{g^{\prime} (s)\,|\, s\in {{\mathbb{R}}}_{+}\}$, and also map *ω*_*n***k**_ to *s*^−1^*ω*_*n***k**_ and DOS(*ω*) to *s*DOS(*sω*); i.e., also leaving the **y**-labels invariant.

Of ${{{{{{{{\mathcal{G}}}}}}}}}_{{{{{{{{\rm{s}}}}}}}}}$ and ${{{{{{{{\mathcal{G}}}}}}}}}_{{{{{{{{\rm{s}}}}}}}}}^{\prime}$, only the amplitude-scaling ${{{{{{{{\mathcal{G}}}}}}}}}_{{{{{{{{\rm{s}}}}}}}}}$ is pixel-compatible (${{{{{{{{\mathcal{G}}}}}}}}}_{{{{{{{{\rm{s}}}}}}}}}^{\prime}$ can be implemented as a tiling-operation in the unit cell, which, however requires down-sampling). Accordingly, we restrict our focus to the pixel-compatible invariances of the product group ${{{{{{{\mathcal{G}}}}}}}}={{{{{{{{\mathcal{G}}}}}}}}}_{0}\times {{{{{{{{\mathcal{G}}}}}}}}}_{{{{{{{{\bf{t}}}}}}}}}\times {{{{{{{{\mathcal{G}}}}}}}}}_{{{{{{{{\rm{s}}}}}}}}}$ and sampled its elements randomly. In practice, the sampling-frequency of each element in ${{{{{{{\mathcal{G}}}}}}}}$ is a hyperparameter of the pre-training stage (see Methods and SI section S5).

### Prediction of PhC DOS

To assess the trained network’s performance in an easily interpretable setting, we define the evaluation error metric, following ref. ^62^:

4 $${{{{{{{{\mathcal{L}}}}}}}}}^{{{{{{{{\rm{eval}}}}}}}}}=\frac{{\sum }_{\omega /{\omega }_{0}}\left|\right.{{{{{{{{\rm{DOS}}}}}}}}}_{{{\Delta }}}^{{{{{{{{\rm{pred}}}}}}}}}-{{{{{{{{\rm{DOS}}}}}}}}}_{{{\Delta }}}\left|\right.}{{\sum }_{\omega /{\omega }_{0}}{{{{{{{{\rm{DOS}}}}}}}}}_{{{\Delta }}}},$$

where ${{{{{{{{\rm{DOS}}}}}}}}}_{{{\Delta }}}={S}_{{{\Delta }}}* {{{{{{{\rm{DOS}}}}}}}}={\omega }_{0}^{-1}{{{{{{{\bf{y}}}}}}}}+{{{{{{{{\rm{DOS}}}}}}}}}_{{{{{{{{\rm{EL}}}}}}}}}$ and ${{{{{{{{\rm{DOS}}}}}}}}}_{{{\Delta }}}^{{{{{{{{\rm{pred}}}}}}}}}={\omega }_{0}^{-1}{{{{{{{{\bf{y}}}}}}}}}^{{{{{{{{\rm{pred}}}}}}}}}+{{{{{{{{\rm{DOS}}}}}}}}}_{{{{{{{{\rm{EL}}}}}}}}}$ are the true and predicted *S*_Δ_-smoothened DOS, respectively, and the sums are over the spectral range 0.24 ≤ *ω*/*ω*_0_ ≤ 0.96 (we omit the spectral region 0 ≤ *ω*/*ω*_0_ < 0.24 during evaluation to get a more critical metric, since the DOS has no significant features there). The network architecture and training details (loss functions, hyperparameters, layers etc.) are discussed in the Methods section.

**Fig. 4:**
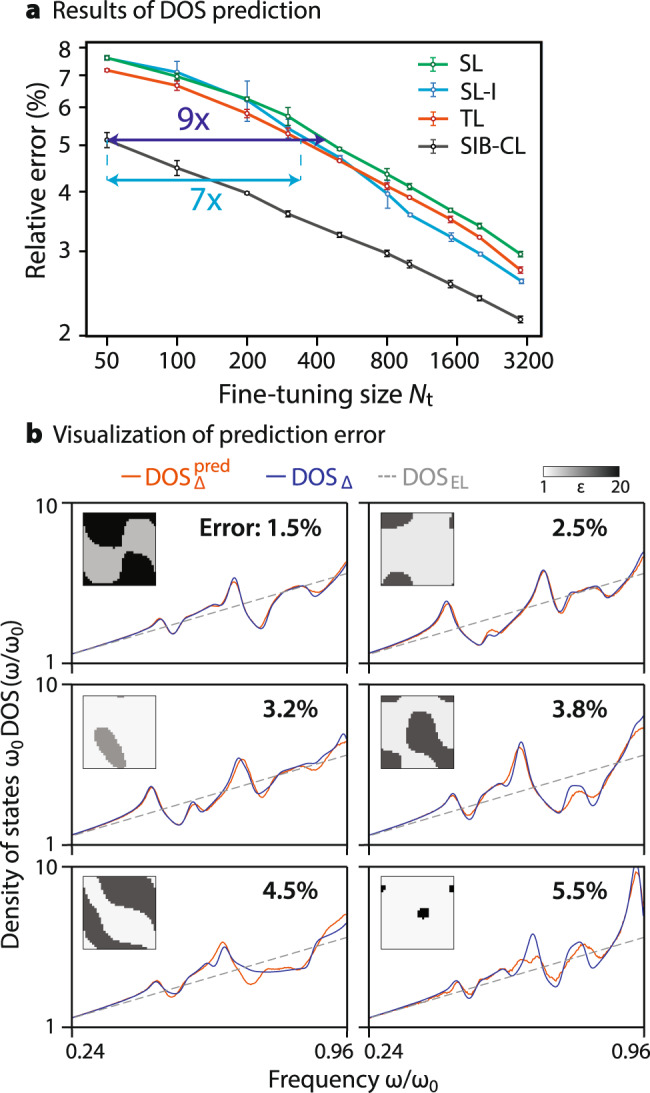
Network prediction results for PhC-DOS problem. **a** Network prediction error against fine-tuning dataset sizes, *N*_t_, between 50 and 3000, when using our SIB-CL framework (based on SimCLR^53^ for contrastive learning) compared against the baselines: direct supervised learning (SL) and standard approaches involving transfer learning (TL) or involving invariances via data augmentation (SL-I). A 9-fold (7-fold) reduction in target data requirements is obtained by using SIB-CL over SL (SL-I, TL) at a relative error target of ~5.1%. Error bars show the 1*σ* uncertainty level estimated from varying the data selection of *D*_t_. **b** Examples of the DOS spectrum predicted by the SIB-CL-trained network compared against the actual DOS at various error levels (insets depict associated unit cells, shown here using the network-inputs' resolution of 32 × 32).

The performance of SIB-CL under this error measure is evaluated in Fig. 4 and contrasted with the performance of the baselines. In practice, to minimize the fluctuations due to sample selection, we show the mean of ${{{{{{{{\mathcal{L}}}}}}}}}^{{{{{{{{\rm{eval}}}}}}}}}$ for three separate fine-tuning stages on distinct randomly-selected datasets of size *N*_t_, evaluated on a fixed test set.

A significant reduction of prediction error is observed for SIB-CL over the baselines, especially for few fine-tuning samples: e.g., at *N*_t_ = 100, SIB-CL has 4.6% error while SL, SL-I and TL have 7.6, 7.1 and 6.9% error respectively. More notably, we see a large reduction in the number of fine-tuning samples *N*_t_ needed to achieve the same level of prediction error, which directly illustrates the data savings in the data-scarce problem. We obtain up to 9×(7×) savings in *N*_t_ when compared to SL (SL-I or TL) at a target prediction error of ~5.1%. These savings highlight the effectiveness of SIB-CL over simple supervised learning (SL) as well as techniques leveraging a single source of auxiliary information, here represented by surrogate-based TL or invariance-augmented SL (SL-I). The predicted and true DOS are compared as functions of frequency in Fig. 4b across a range of error levels as realized for different unit cell input. Further, SIB-CL also incorporates the *combined* invariance and surrogate information more effectively than is achievable e.g., by incorporating the invariance information as data augmentation in surrogate-based transfer learning (invariance-augmented transfer learning, TL-I). This is demonstrated by Table 1, where a steady performance advantage of SIB-CL over TL-I is observed.

**Table 1:** PhC-DOS prediction.

	*N*_*t*_ = 500	*N*_*t*_ = 1000	*N*_*t*_ = 3000
TL-I	3.40 ± 0.06	2.98 ± 0.03	2.38 ± 0.02
SIB-CL	3.25 ± 0.04	2.82 ± 0.05	2.16 ± 0.03

Comparing SIB-CL with a simple, invariance-augmented transfer learning (TL-I) approach.

Both techniques incorporate the same level of auxiliary information, varying only in their learning algorithm. Values give the network prediction error (in %) at different fine-tuning dataset sizes *N*_t_.

A strong motivation for exploring deep learning methods in scientific predictive modeling is to accelerate design processes, since trained neural networks are able to offer cheap evaluations of the target problem; it is thus instructive to assess the computational savings of a trained network. For the DOS problem, the inference time of our trained neural network takes 0.005s on a single Intel Xeon Gold 6148 CPU core, while the traditional numerical method takes 14.5s for a single photonic crystal on the same hardware, resulting in a ≈ 3000 factor speed up. Such savings are highly significant, particularly for design optimization applications where a huge number of forward predictions are often necessary.

To demonstrate that the effectiveness of SIB-CL extends beyond the DOS prediction problem, we also trained a network using SIB-CL and all baselines to predict the PhC band structure (see SI section S2). For this task, the network labels **y** are *ω*_*n***k**_/*ω*_0_, sampled over a 25 × 25 **k**-grid and over the first 6 bands, i.e., ${{{{{{{\bf{y}}}}}}}}\in {{\mathbb{R}}}_{\ge 0}^{6\times 25\times 25}$, while the input labels **x** remain unchanged. Unlike the DOS, the band structure is not invariant under the elements of ${{{{{{{{\mathcal{G}}}}}}}}}_{0}$, but remains invariant under translations (${{{{{{{{\mathcal{G}}}}}}}}}_{{{{{{{{\bf{t}}}}}}}}}$) and refractive amplitude scaling (${{{{{{{{\mathcal{G}}}}}}}}}_{{{{{{{{\rm{s}}}}}}}}}$), i.e., ${{{{{{{\mathcal{G}}}}}}}}={{{{{{{{\mathcal{G}}}}}}}}}_{{{{{{{{\bf{t}}}}}}}}}\times {{{{{{{{\mathcal{G}}}}}}}}}_{{{{{{{{\rm{s}}}}}}}}}$. Also for this task, we found SIB-CL to enable significant data savings, ranging up to 60× relative to the SL baseline.

### Data generation for 3D Schrödinger equation

As a test of the applicability of SIB-CL to higher-dimensional problems, we consider predicting the ground state energies of the single-particle, time-independent Schrödinger equation (TISE) for random 3D potentials in box. This problem demonstrates a proof-of-principle application to the domain of electronic structure calculations, which is of fundamental importance to the understanding of molecules and materials across physics, chemistry, and material science.

**Fig. 5:**
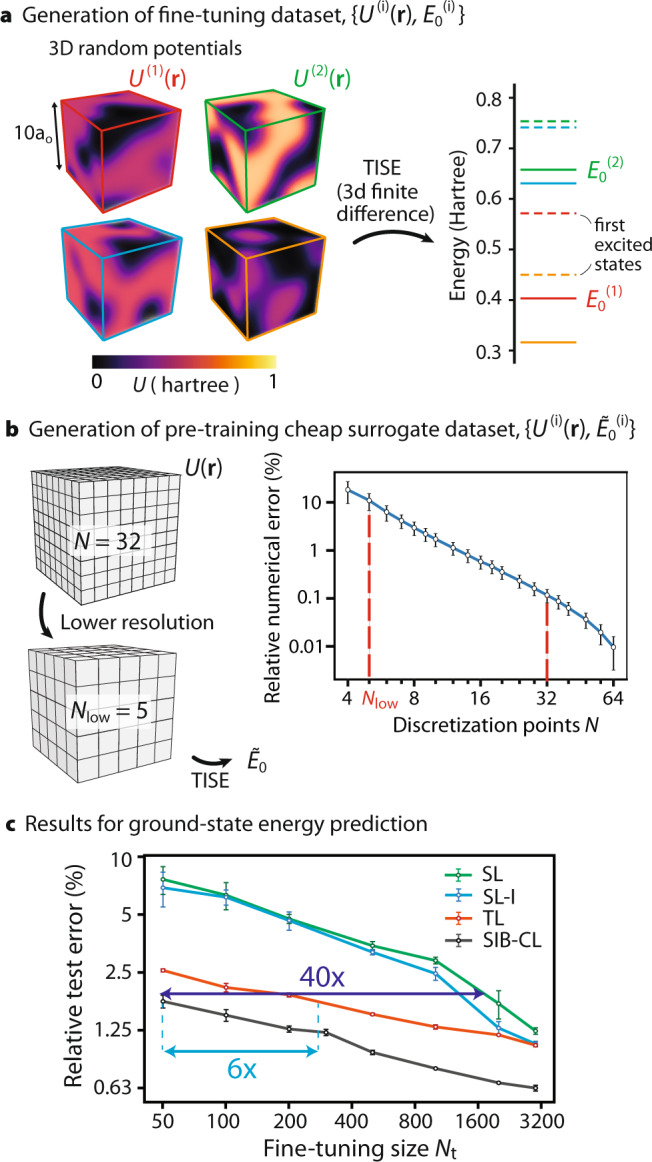
3D Time-independent Schrödinger Equation (TISE). **a** 3D random `in-a-box' potentials and their associated ground-state energies, *E*_0_. **b** Left: The surrogate dataset was generated by reducing the input unit cell resolution from 32 × 32 × 32 to 5 × 5 × 5. Right: The relative numerical error of *E*_0_ at various discretization points *N* when compared to *N* = 128 (the `theoretical' value) shows that *N* = 5 (*N* = 32) is a good choice for the reduced (original) resolution since it gives a relatively high (low) error of 10% (0.1%). Error bars show 1*σ* uncertainty over 10 random samples. **c** Network prediction error against fine-tuning dataset sizes *N*_t_ for SIB-CL and the baselines on the TISE problem. The plot for SIB-CL shows the best results among the two contrastive learning techniques, SimCLR^53^ and BYOL^54^. Error bars show the 1*σ* uncertainty when varying dataset selection. SIB-CL was seen to give up to 40×(6×) data savings when compared to SL (TL).

The eigenstates *ψ*_*n*_ and eigenenergies *E*_*n*_ of a (non-relativistic) single-particle electron in a potential *U*(**r**) are the eigensolutions of the TISE:

5 $$\hat{H}{\psi }_{n}=(\hat{T}+\hat{U}){\psi }_{n}={E}_{n}{\psi }_{n},$$

where $\hat{H}=\hat{T}+\hat{U}$ is the Hamiltonian consisting of kinetic $\hat{T}=-\frac{1}{2}{\nabla }^{2}$ and potential energy $\hat{U}=U({{{{{{{\bf{r}}}}}}}})$ contributions. Here, and going forward, we work in Hartree atomic units (h.a.u.). For simplicity, we consider random potentials *U*(**r**) confined to a cubic box of side length 10 Bohr radii (*a*_0_), with values in the range [0, 1] Hartree (see Methods for details). Examples of the generated potentials are shown in Fig. 5a (left).

We associate the network input–label pairs (**x**, *y*) with the potentials *U*(**r**) (sampled over a 32 × 32 × 32 equidistant grid) and ground state energies *E*_0_, respectively. We evaluate *E*_0_ by using (central) finite differences with implicit Dirichlet boundary conditions to discretize Eq. (5), which is subsequently solved using an iterative sparse solver^68^. The target dataset *D*_t_ is computed using a 32 × 32 × 32 finite-differences discretization, with an estimated mean numerical error ≈0.1% (Fig. 5b, left).

In the previously considered PhC DOS problem, the surrogate dataset *D*_s_ was built from a particularly simple input class with exact and inexpensive labels. Here, instead, we assemble *D*_s_ by including the original range of inputs **x** but using approximate labels $\tilde{y}$. In particular, we populate the surrogate dataset with input–label pairs $({{{{{{{\bf{x}}}}}}}},\tilde{y})$, with $\tilde{y}={\tilde{E}}_{0}$ computed from a low-resolution finite-difference 5 × 5 × 5 discretization of *U*(**r**) (Fig. 5b). ${\tilde{E}}_{0}$ has a relatively high error of ~10% (Fig. 5b, right) but is orders of magnitude faster to compute: e.g., a naive power iteration eigensolver requires *O*(*n*^2^) operations per iteration (with $n=N^{3}$ denoting the number of grid-voxels and *N* the grid-points per dimension), such that iterations at *N* = 5 require ~ 10^5^-fold less work than at *N* = 32.

To assess the impact of the choice of surrogate data, we also examine an alternative surrogate dataset, with input-label pairs $(\tilde{{{{{{{{\bf{x}}}}}}}}},\tilde{y})$, derived from quantum harmonic oscillator (QHO) potentials:

6 $$\tilde{{{{{{{{\bf{x}}}}}}}}}=\tilde{U}({{{{{{{\bf{r}}}}}}}})=\frac{1}{2}{{{{{{{{\boldsymbol{\omega }}}}}}}}}^{\circ 2}\cdot {({{{{{{{\bf{r}}}}}}}}-{{{{{{{\bf{c}}}}}}}})}^{\circ 2},$$

where ${({{{{{{{{\bf{A}}}}}}}}}^{\circ n})}_{i}={A}_{i}^{n}$ is the Hadamard (element-wise) power operator. We define the associated surrogate labels by the open-boundary QHO energies, i.e., by $\tilde{y}={\tilde{E}}_{0}=\frac{1}{2}{\sum }_{i}{\omega }_{i}$, and assign the input $\tilde{{{{{{{{\bf{x}}}}}}}}}$ by the in-box grid discretization of $\tilde{U}({{{{{{{\bf{r}}}}}}}})$. The $\tilde{y}$ labels consequently reflect an example of analytically approximated labels (here, with approximation-error due to the neglect of the Dirichlet boundary conditions); see SI section S3. For quicker training of the network, we use the 2D version of the TISE with this surrogate dataset (i.e., *D*_*s*_ and *D*_*t*_ consist of 2D QHO potentials and 2D random potentials respectively).

### Prediction of the ground-state energy of 3D Schrödinger equation

The ground-state energy is invariant under elements of the symmetry point group, i.e., ${{{{{{{\mathcal{G}}}}}}}}={{{{{{{{\mathcal{G}}}}}}}}}_{0}$ in 2D. In 3D, we instead have the m$\overline{3}$m point group, which notably has 48 elements (instead of just 8 in ${{{{{{{{\mathcal{G}}}}}}}}}_{0}$).

Figure 5c shows the results using the surrogate dataset of reduced resolution data, compared against the baselines. We observe up to 40 × data savings for SIB-CL when compared to SL. Additionally, consistently with our PhC experiments in Table 1, SIB-CL also here outperforms invariance-augmented TL (SI section S3). As a validation step, the prediction accuracies are noted to be in the orders of ≈1%, making the surrogate (target) dataset with ≈10% (≈0.1%) numerical error an appropriate design choice as approximate (target) data. For the experiments using the QHO surrogate dataset, we obtain up to 4 × savings when using SIB-CL compared to SL (see SI section S3); the data savings are diminished, within expectations, since the QHO dataset is way simpler and contains less information to transfer.

## Discussion

The widespread adoption and exploitation of data-driven techniques, most prominently deep learning via neural networks, to scientific problems has been fundamentally limited by a relative data scarcity. That is, data is only rarely available in the quantities required to train a network to faithfully reproduce the salient features of nontrivial scientific problems; moreover, even if such data can be generated, it typically requires excessive computational effort. Here, we have introduced SIB-CL, a framework that overcomes these fundamental challenges by incorporating prior knowledge and auxiliary information, including problem invariances, “cheap” problem classes, and approximate labels. With SIB-CL, the required quantities of costly, high-quality training data is substantially reduced, opening the application-space of data-driven techniques to a broader class of scientific problems.

We demonstrated the versatility and generality of SIB-CL by applying it to problems in photonics and electronic structure, namely to the prediction of the DOS and band structures of 2D PhCs and the ground state energies of the TISE. Through our experiments, we demonstrated that even very simple sources of auxiliary information can yield significant data savings. For instance, the group of invariances ${{{{{{{\mathcal{G}}}}}}}}$ can be just a set of simple rotations and mirrors as in the TISE problem. Similarly, there are diverse options for constructing the surrogate dataset: here, we explored the use of simplified structures where (semi-) analytical solutions exist (e.g., circular structures of PhCs), approximate calculations of the target problem (e.g., reduced resolution computations of TISE), and even a combination of the two (e.g., approximated energies of QHO potentials in the TISE problem). Most natural science disciplines, especially physics, have deep and versatile caches of such approximate and analytical approaches which can be drawn from to create suitable surrogate datasets.

In the problems studied here, SIB-CL outperformed *all* baselines (including invariance-augmented baselines, see SI section S4). We conjecture that SIB-CL’s performance advantage stems predominantly from enforcing problem invariances via a contrastive loss, which is more effective than naive data augmentation (cf. the performance edge of SIB-CL over TL-I). To substantiate this hypothesis, we performed several ablation experiments (see SI Section S5). Firstly, when all invariance information are disregarded in SIB-CL (i.e., if the group of invariances ${{{{{{{\mathcal{G}}}}}}}}$ is reduced to the trivial identity group), we observe very similar performance to TL. This demonstrates that the constrastive stage is only effective in combination with invariance information, or, equivalently, that the utility of the contrastive stage hinges strongly on attracting nontrivial positive pairs rather than merely repelling negative pairs.

Next, we explored the consequences of selectively and increasingly removing invariances from ${{{{{{{\mathcal{G}}}}}}}}$. We found that including more invariances strictly improves SIB-CL’s performance, consistent with expectations since the elements of ${{{{{{{\mathcal{G}}}}}}}}$ are *true* invariances of the downstream task. This is contrary to the standard self-supervised learning (SSL) paradigm which is task-agnostic, i.e., the downstream task is not known during the contrastive learning stage, and transformations may not be *true* invariances of the downstream problem so including more transformations can sometimes be harmful^49,53,69^. To determine the relative and combined efficacy of SIB-CL’s usage of transfer learning and contrastive learning, we performed ablation experiments (see SI section S5). From these experiments, we establish both the constrastive and transfer learning stages contribute significantly to the accuracy of SIB-CL, with the constrastive stage being the dominant contribution. Pre-training has currently become a standard approach in deep learning with TL and SSL being popular instantiations. TL relies on labeled data leading to task specification while SSL relies on unlabeled data and auxiliary pretext tasks to derive representations that generalize well due to its non-specificity to any particular task. The effectiveness of SIB-CL stems from combining desirable features from each of these pre-training techniques into a coherent framework. More concretely, the use of TL alone may lead to undesirable overfitting to the simplified surrogate labels; concurrently, the higher-dimensional nature of labels common in scientific applications may render SSL techniques to be ineffective if transformations provided via pretext tasks does not provide sufficient context to learn the final predictive task. The combination of unsupervised objectives and end-task objectives can lead to improved performance and data efficiency^70^; this is further exemplified in SI Section S5, showing the gains of SIB-CL over its individual components.

While contrastive learning has gained enormous popularity in recent years, its techniques has mainly found applications in computer vision tasks (e.g., image classification on ImageNet^10^) while its utility to regression problems has remained largely unexplored. Techniques like SimCLR are based on instance discrimination, i.e., the network is trained to discriminate between negative pairs in the batch. Intuitively, such techniques may seem less well-suited to regression problems where the latent space is often continuous rather than discrete or clustered as in classification problems. Indeed, we made several empirical observations that disagree with the findings of standard contrastive learning applications on classification problems. Notably, it is widely corroborated^53,71,72^ that using a larger batch size is always more beneficial, which can be interpreted as the consequence of having more negative pairs for instance discrimination. This empirical finding was not echoed in our experiments, thus suggesting that instance discrimination may not be highly appropriate in regression problems. Motivated by this, we also explored the BYOL technique^54^ which is not based on instance discrimination and does not use explicit negative pairs in its loss function (see SI section S1), but found no performance advantage. Despite many empirical successes, SSL methods remains poorly understood and lacks a solid theoretical explanation^49,73–75^ for why and when these algorithms work well. Our work further underscores and motivates the need to develop such an improved foundation, not only to address the noted deviations from expectations but also to guide the emerging application of contrastive learning techniques to regressions tasks.

Exploiting prior knowledge of symmetries and physical insights has shown to be highly effective for deep learning in the scientific domain. For instance, architectures with hand-crafted or analytically formulated components are commonly used in molecules to invoke chemically meaningful inductive biases or to respect quantum–mechanical properties^30,31,33,76^. There also exists a growing body of work on equivariant networks for various symmetry groups^35–37,77^, particularly for applications in the natural sciences^40,78^, of which our work is highly complementary to. These works are mainly motivated by the fact that the exploitation of symmetry or physical insights provides a strong inductive bias, which constrains the space of possible models or allow it to properly model physical limits, ultimately achieving better predictive accuracy and higher data efficiency. Like these networks, SIB-CL also aims to create a network that exploits underlying symmetries and known physical invariances of the problem. However, rather than hard-coding invariance information into the model architecture, the process is implemented/achieved organically via contrastive learning. The price paid for this more generic approach, is that feature invariance to the symmetry group ${{{{{{{\mathcal{G}}}}}}}}$ is only approximately achieved—to a degree expressed indirectly by the NT-Xent loss (Eq. (1))—rather than exactly as is the case for hard-coded problem-specific architectures. Conversely, SIB-CL has the advantage of being simple and readily generalizable to *any* known invariance, i.e., requires no specialized kernels or mathematical construction, and can readily incorporate additional invariances without changes to the underlying architecture. Given the ubiquity and impact of symmetry-preserving equivariant architectures in scientific deep learning, we compared SIB-CL against one prominent architecture in this domain, namely the E(2)-equivariant CNNs proposed by Weiler and Cesa^79^ (see SI section S7). Our experiments show that SIB-CL remains competitive, and even outperforms, such equivariant architectures. Relatedly, SIB-CL’s superior performance over TL-I (Table 1) similarly suggests that using contrastive learning to enforce invariances is likely to be more effective than naive data augmentation.

Our work provides insights on how issues of data scarcity can be overcome by leveraging sources of auxiliary information in natural science problems. The SIB-CL framework presented in this work demonstrates how such auxiliary information can be readily and generically incorporated in the network training process. Our work also provides insights on the thus-far less-explored application of contrastive learning for regression tasks, opening up opportunities for applications in several domains dominated by regression problems, in particular, the natural sciences. Finally, we note that SIB-CL was developed with the motivation that many problems in the natural sciences are endowed with approximate or analytical approaches that can be used to create a surrogate dataset at low computation cost and thus are limited to domains as such.

## Methods

### PhC unit cells and DOS processing

We parameterize *ε*(**r**) by choosing a level set of a Fourier sum function *ϕ*, defined as a linear sum of plane waves with frequencies evenly spaced in the reciprocal space (up to some cut-off). i.e.,

7 $$\phi ({{{{{{{\bf{r}}}}}}}})=\,{{\mbox{Re}}}\,\left[\mathop{\sum }\limits_{k=1}^{9}{c}_{k}\exp (2\pi {{{{{{{\rm{i}}}}}}}}{{{{{{{{\bf{n}}}}}}}}}_{k}\cdot {{{{{{{\bf{r}}}}}}}})\right],$$

where each **n**_*k*_ is a 2D vector (*n*_*x*_, *n*_*y*_) and we used 3 Fourier components per dimension, i.e., *n*_*x*_, *n*_*y*_ ∈ [ − 1, 0, 1] (and thus the summation index *k* runs over 9 terms). *c*_*k*_ is a complex coefficient, *c*_*k*_ = *re*^i*θ*^ with *r*, *θ* separately sampled uniformly in [0, 1). Finally, we uniformly sample a filling fraction, defined as the fraction of area in the unit cell occupied by *ε*_1_, in [0, 1) to determine the level set Δ so as to obtain the permittivity profile:

8 $$\varepsilon ({{{{{{{\bf{r}}}}}}}})=\left\{\begin{array}{ll}{\varepsilon }_{1}&\phi ({{{{{{{\bf{r}}}}}}}})\,\le \,{{\Delta }}\\ {\varepsilon }_{2}&\phi ({{{{{{{\bf{r}}}}}}}}) \, > \, {{\Delta }}\end{array}\right..$$

This procedure produces periodic unit cells with features of uniformly varying sizes due to the uniform sampling of the filling ratio and without strongly divergent feature scales thus corresponding to fabricable designs.

With the MIT Photonic Bands (MPB) software^63^, we use 25 × 25 plane waves (and also a 25 × 25 **k**-point resolution) over the Brillouin zone −*π*/*a* < *k*_*x*,*y*_ ≤ *π*/*a* to compute the band structure of each unit cell up to the first 10 bands and also extract the group velocities at each **k**-point. We then computed the DOS for *ω*/*ω*_0_ ∈ [0, 0.96] over 16000 equidistantly-spaced frequency samples using the generalized Gilat–Raubenheimer (GGR) method^61,62^. Next, we computed the *S*_Δ_-smoothened DOS, i.e., ${{{\mathrm{DOS}}}}_{\Delta}=S_{\Delta} * {{{\mathrm{DOS}}}}$, using a Gaussian filter ${S}_{{{\Delta }}}(\omega )={{{{{{{{\rm{e}}}}}}}}}^{-{\omega }^{2}/2{{{\Delta }}}^{2}}/\sqrt{2\pi }{{\Delta }}$ of spectral width Δ = 0.006*ω*_0_. Before defining the associated network labels **y**, we downsampled DOS_Δ_ to 400 frequency points. Finally, the network **y**-labels are constructed according to Eq. (3), i.e., by subtracting the background “empty-lattice” DOS—i.e., ${{{{{{{{\rm{DOS}}}}}}}}}_{{{{{{{{\rm{EL}}}}}}}}}(\omega )={a}^{2}{n}_{{{{{{{{\rm{avg}}}}}}}}}^{2}\omega /2\pi {c}^{2}$, the DOS of a uniform unit cell Ω of index ${n}_{{{{{{{{\rm{avg}}}}}}}}}=\frac{1}{| {{\Omega }}| }{\int}_{{{\Omega }}}n({{{{{{{\bf{r}}}}}}}})\,{{{{{{{{\rm{d}}}}}}}}}^{2}{{{{{{{\bf{r}}}}}}}}$—and rescaling by *ω*_0_. Subtracting DOS_EL_ removes a high-frequency bias during training and was found to improve overall network accuracy.

### 3D TISE unit cells

To generate samples of *U*(**r**), we follow the same procedure in Eqs. (7) and (8) to first create two-tone potential profiles in 3D, i.e., **r** = (*x*, *y*, *z*) and **n**_*k*_ = (*n*_*x*_, *n*_*y*_, *n*_*z*_) are now 3D vectors. We create finer features by increasing the number of Fourier components to *n*_*x*_, *n*_*y*_, *n*_*z*_ ∈ [−2, −1, 0, 1, 2] (and hence the summation in Eq. (7) now runs over 125 terms). We also modify the range of potential, i.e., *ε*_1_ in Eq. (8) is set to 0, while *ε*_2_ is uniformly sampled in [0, 1]. The periodicity is removed by truncating 20% of the unit cell from *each* edge. A Gaussian filter with a kernel size 8% of the (new) unit cell is then applied to smooth the potential profile and, finally, the unit cells are discretized to a resolution of 32 × 32 × 32. This procedure is illustrated in SI section S3 and is similarly used to produce the 2D unit cells, discretized to 32 × 32, when using the QHO surrogate dataset. The ratio between the length scale and potentials’ dynamic range was also carefully selected to produce non-trivial wavefunctions, so as to create a meaningful deep learning problem (see SI section S3 for further discussion).

### Model architecture

Our encoder network, **H** consists firstly of 3 to 4 convolutional neural network (CNN) layers followed by 2 fully connected (FC) layers, where the input after the CNNs was flattened before being fed into the FC layers. The channel dimensions in the CNN layers and number of nodes in the FC layers vary for the different problems, and are listed in Table 2. For TISE, the CNN layers have 3D kernels to cater for the 3D inputs, while the CNNs for the remaining problems uses regular 2D kernels used in standard image tasks. For the predictor network, **G**, we used 4 FC layers for all the problems, with number of nodes listed in Table 3. The predictor network for the band structure problem consists of 6 blocks of the same layer architecture, each block leading to each of the 6 bands and separately updated using the loss from each band during training. A similar architecture was used in previous work^5^. We included BatchNorm^80^, ReLU^81^ activations and MaxPooling between the CNN layers, and ReLU activations between all the FC layers in **H** and **G**. For the projector network **J**, we used 2 FC layers with hidden dimension 1024 and ReLU activation between them; the final metric embeddings have dimension 256. **J** is fixed across all problems. Using the DOS prediction problem, we also experimented with deeper projector networks (i.e., increasing to 4 FC layers with the same hidden dimensions), as well as including BatchNorm between the layers, and found small improvements.

**Table 2:** Network architecture for **H**.

Problem	Channel dim per CNN layer	# nodes per FC layer
DOS	[64, 256, 256] (2D)	[1024, **1024**]
Band structure	[64, 256, 256] (2D)	[256, **1024**]
TISE	[64, 256, 256, 256] (3D)	[256, **256**]

Bold values indicate the dimension of the representation for the different problems.

**Table 3:** Network architecture for **G**.

Problem	# nodes per FC layer
DOS	[1024, 1024, 512, **400**]
Band structure	[256, 512, 512, **625**] × 6
TISE	[256, 256, 32, **1**]

Bold values indicate the dimension of the network output which matches the label dimension for that problem.

### Invariance sampling during contrastive learning

In conventional contrastive learning applications in computer vision (CV), different instances of the input are often created via a pre-optimized, sequential application of various data augmentation strategies such as random cropping, color distortion, and Gaussian blur^53,54^. Adopting this technique, we also apply transformations from each sub-group of ${{{{{{{\mathcal{G}}}}}}}}$ in the randomly determined order $[{{{{{{{{\mathcal{G}}}}}}}}}_{{{{{{{{\bf{t}}}}}}}}},{{{{{{{{\mathcal{G}}}}}}}}}_{0},{{{{{{{{\mathcal{G}}}}}}}}}_{{{{{{{{\rm{s}}}}}}}}}]$ and, additionally, experimented various algorithms for performing contrastive learning; see SI Section S5. We find that introducing stochasticity in transformation application is an effective strategy and thus use it in SIB-CL. More specifically, for each sub-group ${{{{{{{{\mathcal{G}}}}}}}}}_{\alpha }$, with *α* ∈ {0, **t**, s}, we set a probability *p*_*α*_ to which any non-identity transformation is applied. (Equivalently, inputs are not transformed with probability (1 − *p*_*α*_).) {*p*_*α*_} is a set of hyperparameters that are often intricately optimized for in standard CV applications (among other hyperparameters such as the order and strength of augmentations); here, for simplicity, we omitted this optimization step. We set *p*_*α*_ = 0.5 for all *α*’s, and sampled the elements uniformly, i.e., each transformation in ${{{{{{{{\mathcal{G}}}}}}}}}_{\alpha }$ is applied with probability 0.5/*m*_*α*_ with *m*_*α*_ being the total number of non-identity elements in ${{{{{{{{\mathcal{G}}}}}}}}}_{\alpha }$.

### PhC DOS prediction loss functions

In step *b* of the pre-training stage where we trained using supervised learning loss on *D*_s_ (Fig. 2b), we used the pre-training loss function

9 $${{{{{{{{\mathcal{L}}}}}}}}}^{PT}={{{{{{{{\rm{mean}}}}}}}}}_{(\omega /{\omega }_{0})}\left(\log (1+| {{{{{{{{\bf{y}}}}}}}}}^{{{{{{{{\rm{pred}}}}}}}}}-{{{{{{{\bf{y}}}}}}}}| )\right),$$

for each sample in the batch, where **y**^pred^ and **y** are the network prediction and the true label of that sample respectively and ∣ ⋅ ∣ gives the element-wise absolute value. We take the mean over the (normalized) frequency axis (*ω*/*ω*_0_) to get a scalar for ${{{{{{{{\mathcal{L}}}}}}}}}^{PT}$. This loss function was used during pre-training (for SIB-CL and the TL baselines); its purpose is to encourage the network to learn from the surrogate dataset the general features in the DOS spectrum and underemphasize the loss at places where the DOS diverges, i.e., at the Van Hove singularities. In our experiments, we found that ${{{{{{{{\mathcal{L}}}}}}}}}^{PT}$ indeed gave better prediction accuracies than the standard L1 or mean squared error (MSE) loss functions. After the pre-training step, the standard L1 loss function was used during fine-tuning on *D*_t_ (Fig. 2c) for SIB-CL and all the baselines.

### PhC band structure prediction loss functions

During supervised training (for both pre-training and fine-tuning), we use the MSE loss function; for evaluation, we use a relative error measure (for easier interpretation) given by,

10 $${{{{{{{{\mathcal{L}}}}}}}}}^{{{{{{{{\rm{eval}}}}}}}}}={{{{{{{{\rm{mean}}}}}}}}}_{{{{{{{{\bf{k}}}}}}}}}\left(\frac{1}{6}\mathop{\sum }\limits_{n=1}^{6}\frac{| {\omega }_{n}^{{{{{{{{\rm{pred}}}}}}}}}({{{{{{{\bf{k}}}}}}}})-{\omega }_{n}({{{{{{{\bf{k}}}}}}}})| }{{\omega }_{n}({{{{{{{\bf{k}}}}}}}})}\right),$$

where *ω*_*n*_(**k**) are the eigen frequencies indexed over band numbers *n* = 1, 2, ..., 6 and **k** are the wave vectors restricted to the Brillouin zone, i.e., −*π*/*a* < *k*_*x*,*y*_ ≤ *π*/*a*. The evaluation loss is taken as the mean over all 6 bands and over all (25 × 25) **k**-points.

### Ground-state energy prediction loss functions

The MSE loss function is similarly used during both the pre-training and fine-tuning stages of supervised training of the ground-state energy prediction problem. During evaluation, we use a simple relative error measure,

11 $${{{{{{{{\mathcal{L}}}}}}}}}^{{{{{{{{\rm{eval}}}}}}}}}=| {y}^{{{{{{{{\rm{pred}}}}}}}}}-y| /y,$$

where *y*^pred^ is the network prediction and *y* = *E*_0_ is the recorded ground-state energy, for each sample in the test set.

### Training hyperparameters

For training the networks in all problems, we used Adam optimizers^82^, with learning rates for the different steps specified in Table 4. We also use an adaptive learning rate scheduler for the fine-tuning stage. Even though standard contrastive learning methods implement a cosine annealing scheduler^83^, we found that this was not beneficial for SIB-CL on our problems and thus omitted it. Additionally, in order to prevent networks **H** and **G** from overfitting to the surrogate dataset, we explored various conventional regularization techniques during the pre-training stage, such as weight decay and dropout. We found that these were not beneficial; instead, we used early stopping where we saved the pre-trained model at various epochs and performed the fine-tuning stage on all of them, picking only the best results to use as the final performance. For SIB-CL, the pre-trained model was saved at {100, 200, 400} epochs, and for TL (both with and without invariances), the pre-trained model was saved at {40, 100, 200} epochs. (See SI section S6 for more details on the checkpoint choices). Finally, another important hyperparameter in our experiments is the kernel size (*n*_*k*_) of the CNN layers; apart from optimizing the learning process, this hyperparameter can be used to adjust the network size. This is important in our experiments since we are training/fine-tuning on varying sizes *N*_t_ of the target dataset; a smaller (bigger) dataset is likely to need a smaller (bigger) network for optimal results. For the DOS prediction, we varied *n*_*k*_ ∈ {5, 7}; for band structures, *n*_*k*_ ∈ {7, 9, 11} and for TISE, *n*_*k*_ ∈ {5, 7}. The same set of *n*_*k*_ was applied for both SIB-CL and all baselines in every problem. Apart from those mentioned here, SIB-CL involves many other hyperparameters not explored here; see additional comments in SI section S6.

**Table 4:** Set of hyperparameters tuned over in the contrastive learning (CL), pre-training (PT) of **G**, and fine-tuning (FT) steps of SIB-CL.

Problem	CL (step *a*)	PT of G (step *b*)	FT (step *c*)
DOS	*B* ∈ {192, 768}	*B* ∈ {16, 32, 64, 128}	*B* ∈ {16, 32, 64, 128}
	*α* ∈ {10^−5^, 10^−4^, 10^−3^}	*α* ∈ {10^−5^, 10^−4^, 10^−3^}	$\alpha \in \left\{1{0}^{-4},5\,\cdot \,1{0}^{-4}\right.,$
			$$\left.1{0}^{-3},5\,\cdot \,1{0}^{-3}\right\}$$
Bandstructure	*B* ∈ {192, 768}	*B* ∈ {16, 32, 64}	*B* ∈ {16, 32, 64}
	*α* ∈ {10^−4^, 10^−3^}	*α* ∈ {10^−4^, 10^−3^}	*α* ∈ {10^−4^, 10^−3^}
TISE	*B* ∈ {384}	*B* ∈ {32, 64, 128}	*B* ∈ {32, 64, 128}
	*α* ∈ {10^−6^, 10^−5^}	*α* ∈ {10^−5^, 10^−4^}	*α* ∈ {10^−4^, 10^−3^}

The baseline algorithms were also tuned with a similar level of effort; (see SI section S6 for details). The main hyperparameters we varied are the batch size (B) and the learning rates (α).

## Supplementary information

Supplementary Information

## Data Availability

PhC band structures were computed using MPB^63^. DOS calculations were carried out using the GGR method, adapted from the MATLAB implementation in ref. ^62^. Numerical solution of the TISE ground-state energies was implemented in Python using SciPy^84^. The datasets generated in this study and source codes used to generate them are available via the code repository at https://github.com/clott3/SIB-CL^85^.

## Code availability

The neural networks were implemented and trained using the PyTorch framework^86^. All source codes used for training, for both SIB-CL and the baselines presented in this work, are publicly available at https://github.com/clott3/SIB-CL^85^.
