# Supplementary material for: Surrogate- and invariance-boosted contrastive learning for data-scarce applications in science
Source: Nat Commun. 2022 Jul 21;13:4223. doi: 10.1038/s41467-022-31915-y (PMC9304370; doi:10.1038/s41467-022-31915-y)
Supplement: Supplementary file 1 — Supplementary Information [file 41467_2022_31915_MOESM1_ESM.pdf]

SUPPLEMENTARY INFORMATION

Surrogate- and invariance-boosted contrastive learning for data-scarce applications in science

Charlotte Loh,[1] Thomas Christensen,[2] Rumen Dangovski,[1] Samuel Kim,[1] and Marin Soljačić[2]

[1] *Department of Electrical Engineering and Computer Science, Massachusetts Institute of Technology, Cambridge, Massachusetts, USA*
[2] *Department of Physics, Massachusetts Institute of Technology, Cambridge, Massachusetts, USA*

CONTENTS

S1. BYOL illustration & results 1

S2. Photonic Crystals Band Structure Prediction 2

S3. TISE: Additional details and results 3

S4. Additional baselines: including invariance information using data augmentation 4

S5. "Baking in" invariances using contrastive learning: Ablation studies and further experiments 6

S6. Additional comments on hyperparameters 8

S7. Comparison with state-of-the-art equivariant network 9

Supplemental References 12

S1. BYOL ILLUSTRATION & RESULTS

In BYOL [S2], instead of a single network like in SimCLR [S1], there are two networks of the same architecture (Supplementary Figure 1a): a trainable online network (with weights parameterized by θ) and a fixed target network (with weights parameterized by ξ). The two variations of a unit cell (obtained via sampling from $\mathcal{G}$) are separately fed through the online and target network, where the online network features an additional "BYOL predictor" sub-network which is not present in SimCLR. The weights of the online network θ are then updated by minimizing the mean squared error (MSE) between the (normalized) online and target embeddings, as given by,

$$\mathcal{L}^{BYOL}_{jj',\theta\xi} = \left\|\bar{K}(z_{j,\theta}) - \bar{z}_{j',\xi}\right\|^2 = 2 - 2 \cdot \frac{\langle K(z_{j,\theta}), z_{j',\xi}\rangle}{\left\|K(z_{j,\theta})\right\| \cdot \left\|z_{j',\xi}\right\|}, \tag{S1}$$

where $\|\cdot\|$ indicates the L2 norm, K is the "BYOL predictor" and z represent the respective embeddings illustrated in Supplementary Figure 1a. As for the target network, at each training step, it is updated using an exponential moving average of the online network weights,

$$\xi \leftarrow \tau\xi + (1-\tau)\theta, \tag{S2}$$

where $\tau \in [0,1]$ is a target decay rate hyperparameter. Unlike SimCLR's loss function (Eq. (1) in the main text), where negative pairs are explicitly being "pushed apart" due to the denominator of the cross entropy loss function, the BYOL loss (Eq. (S1)) does not explicitly involve negative pairs.

In Supplementary Figure 1b, we compare the results when replacing the SimCLR technique in SIB-CL with the BYOL technique, evaluated on the DOS prediction problem for Photonic Crystals (PhC); we also depict the baselines' performances for comparison. When including the full suite of invariance information (i.e. translations ($\mathbf{t}$), rotations (C), mirrors (σ) and scaling (s)), BYOL was observed to give slightly poorer performance compared to SimCLR. Here, for convenience, rotation and mirror operations are collectively defined as $C = \{C_2, C_4^{\pm}\}$ and $\sigma = \{\sigma_h, \sigma_v, \sigma_d^{(\prime)}\}$ respectively. When removing the scaling transformation, BYOL was seen to give similar results to SimCLR (as well as similar to BYOL including the scaling transformation). This suggests that the use of negative pairs during contrastive learning has no significant implications, despite initial intuition that such a concept may seem inappropriate for regression problems. Additionally, our results also suggest that BYOL was observably not effective at handling the scaling transformation. This observation was further echoed in the TISE ground-state energy prediction

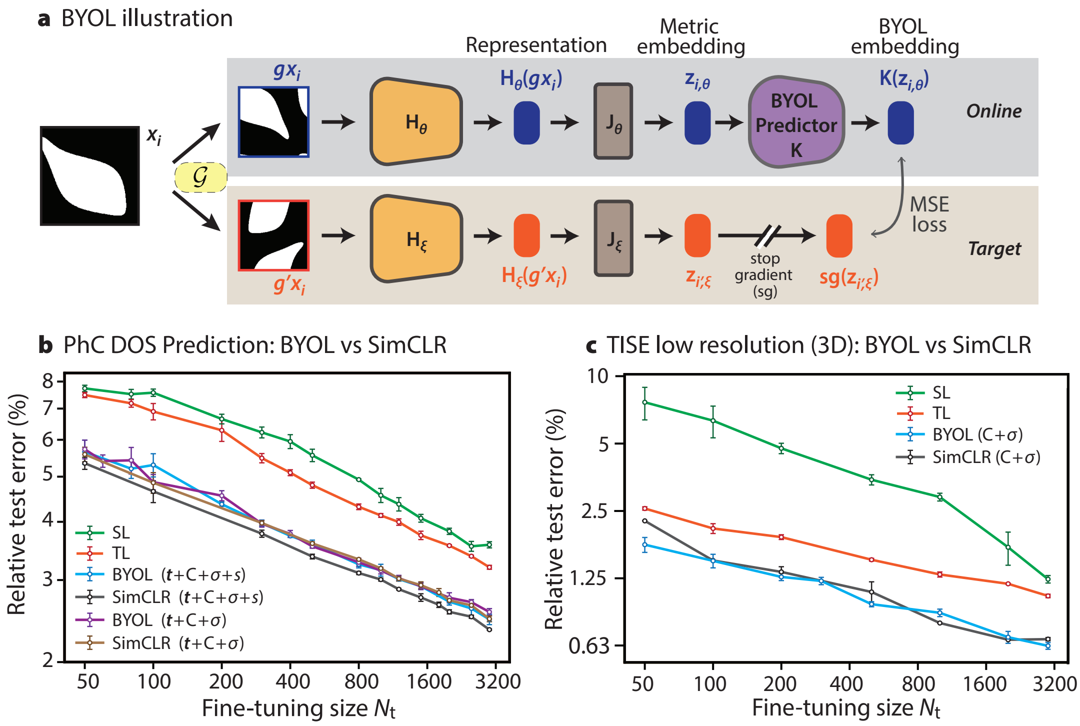

Supplementary Figure 1. BYOL for contrastive learning a) Illustration of the BYOL algorithm, which includes two networks, an online and a target network. The variables here correspond to the variables defined in Fig. 2 in the main text, except here we include explicit reference to the network weights using θ and ξ. **b**) Results for using BYOL in SIB-CL compared against when using SimCLR on the PhC DOS prediction problem, when using various combinations of invariances: translations (**t**), rotations (C) and mirrors (σ), and scaling (s). **c**) Results for using BYOL in SIB-CL compared against when using SimCLR on the ground-state energy prediction problem (in 3D, and using low resolution data as the surrogate dataset). Error bars show the 1σ uncertainty level when varying the data selection of the fine-tuning dataset.

problem (Supplementary Figure 1c); here, the performance differences between the two techniques were observed to mostly occur within the 1σ uncertainty range. In other words, this result implies that neither technique outperforms the other with statistical significance.

S2. PHOTONIC CRYSTALS BAND STRUCTURE PREDICTION

In this section, we describe additional experiments of performing PhC band structure regression, where we trained a network to predict the transverse-magnetic (TM) band structures of the PhCs, up to the first 6 bands. Unlike the DOS, we do not integrate over the Brillouin zone and thus the set of invariant transformations is reduced to just the choice of the unit cell origin (i.e., to translation invariance, $\mathcal{G} = \mathcal{G}_t$). During supervised training (for both pre-training and fine-tuning), we use the mean squared error (MSE) loss function; for evaluation, we use a relative error measure (for easier interpretation) given by,

$$\mathcal{L}^{\text{eval}} = \text{mean}_{\mathbf{k}} \left(\frac{1}{6} \sum_{n=1}^{6} \frac{|\omega_n^{\text{pred}}(\mathbf{k}) - \omega_n(\mathbf{k})|}{\omega_n(\mathbf{k})} \right), \tag{S3}$$

where $\omega_n(\mathbf{k})$ are the eigen frequencies indexed over band numbers $n = 1, 2, ..., 6$ and $\mathbf{k}$ are the wave vectors restricted to the Brillouin zone, i.e. $-\pi/a < k_{x,y} \leq \pi/a$. The evaluation loss is taken as the mean over all 6 bands and over all $\mathbf{k}$-points. Details of the network architecture and training hyperparameters used in these experiments are described in the Methods section of the main text.

Resolution	Band structure error	DOS error
4×4	3.5%	18.7%
8×8	1.7%	3.1%
16×16	0.5%	1.0%

Supplementary Table 1. Computational accuracy of band structure compared to DOS.

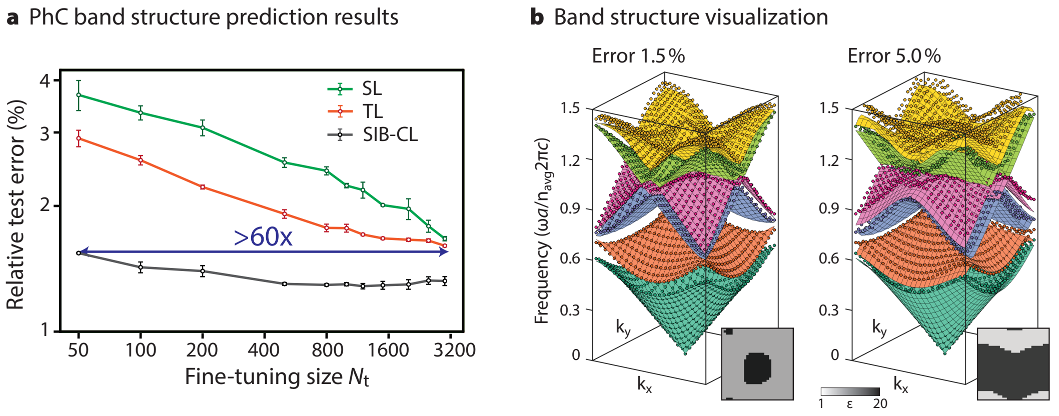

Supplementary Figure 2. PhC band structure prediction a) Performance of SIB-CL (based on SimCLR [S1] for the contrastive learning step), compared against the baselines introduced in the main text, for band structure prediction. Error bars show the 1σ uncertainty level estimated from varying the data selection of the fine-tuning dataset. **b**) Examples of the band structure predicted by the SIB-CL trained network (indicated by markers) compared against the actual band structure (indicated by surface plots) at 1.5% and 5% error levels; insets depict the associated unit cells.

The results are shown in Supplementary Figure 2a, where SIB-CL was observed to give more than 60× data savings compared to any of the baselines. Examples of the band structure predicted by the trained network at various error levels are also depicted in Supplementary Figure 2b, where the markers indicate the network prediction and the surface plots depict the true band structure. While the data savings is highly impressive, we found the choice of the evaluation metric to be more subtle (than the DOS prediction task). This is because most of the unique features in the band structure of each unit cell are contained only at some **k**-points, and the band structure at the remaining **k**-points can be very well approximated using the "empty-lattice" (a featureless unit cell filled entirely by the average permitivitty); thus we undesirably under-emphasize the prediction error when we take the mean over **k**. This is further illustrated in Supplementary Table 1, where we show the relative numerical error (according to metric Eq. (S3)) of the band structures when computed using a reduced resolution (i.e. approaching the "empty-lattice") of the unit cell. At a resolution of 4×4 we have an error amounting to only 3.5%, suggesting that the "empty-lattice" band structure is a rather good approximation. In other words, in order to critically evaluate the band structure prediction problem, an asymmetric loss function (that emphasizes the differences at the "special" **k**-points) should be used. Nevertheless, Supplementary Figure 2a serves as clear evidence that SIB-CL outperforms the baselines (since we use the same evaluation metric in SIB-CL and all baselines). In contrast, the DOS does not have this issue (Supplementary Table 1) and thus it is acceptable to use a straightforward evaluation metric, as presented in the main text.

S3. TISE: ADDITIONAL DETAILS AND RESULTS

The process of generating the random potentials $U(\mathbf{r})$ for the time-independent Schrödinger equation (TISE) problem, as explained in the Methods section of the main text, is illustrated in Supplementary Figure 3a (here in 2D for illustration purposes). When generating the dataset, special care was taken

in choosing the dynamic range of the potential and the physical size of the infinitely-bounded box to ensure that we produce non-trivial solutions. A dataset with trivial solutions would be one where the samples have their wavefunctions either 1) resemble the ground-state solution of a "particle in a box", i.e. the variations in the potential are too weak relative to the confinement energy of the box, or 2) highly localized in local minimas of the potential, i.e. the variations in the potentials are too strong relative to the confinement energy. Both extremes would not create a meaningful deep learning problem and thus we avoid them by carefully controlling the ratio between the potential range and length scale. In Supplementary Figure 3b, we display some examples of potentials in our dataset and their corresponding ground-state solution, and verify that they indeed avoid the two aforementioned extremes. We show them in 2D here for illustration purposes, whereas in the main text the TISE problem is in 3D.

Consistently with the PhC results in the main text, Supplementary Table 2 compares SIB-CL against invariance-augmented TL at various number of labeled samples. While both techniques incorporate the same level of auxiliary information (of both the surrogate dataset and invariance information), SIB-CL was observed to provide significant performance gains.

	$N_t = 200$	$N_t = 1000$	$N_t = 3000$
TL-I	1.92 ± 0.16	1.05 ± 0.05	0.91 ± 0.07
SIB-CL	1.33 ± 0.07	0.79 ± 0.00	0.67 ± 0.01

Supplementary Table 2. TISE ground-state energy prediction. Comparing SIB-CL with a simple, invariance-augmented transfer learning (TL-I) approach. Both techniques incorporate the same level of auxiliary information, varying only in their learning algorithm. Values give the network prediction error (in %) at different fine-tuning dataset sizes N_t.

Apart from using reduced resolution data as the surrogate dataset for the TISE problem as presented in the main text, we also experimented with using a simple, analytically-defined dataset like a dataset of quantum harmonic oscillators (QHO). For quicker training of the neural networks, we work with the TISE problem in 2D for these experiments. Different unit cells in the QHO surrogate dataset are defined using different values of $\boldsymbol{\omega} = (\omega_x, \omega_y)$ and $\mathbf{c} = (c_x, c_y)$ in

$$\tilde{U}(\mathbf{r}) = \tfrac{1}{2}\boldsymbol{\omega}^{\circ 2} \cdot (\mathbf{r} - \mathbf{c})^{\circ 2}, \tag{S4}$$

and the ground-state energy can be analytically defined as $\tilde{E}_0 = \frac{1}{2}(\omega_x + \omega_y)$ (see Supplementary Figure 3c). We experimented with the dynamic ranges of $\boldsymbol{\omega}$ and $\mathbf{c}$ to ensure that the potential of the unit cells are sufficiently large near the infinite boundaries, since our prediction task assumes Dirchlet boundary conditions. To do so, we solved for the ground-state energies of a small sample set of QHO potentials using our eigensolver (with Dirichlet boundary conditions) and chose dynamic ranges of $\boldsymbol{\omega}$ and $\mathbf{c}$ such that the error of the analytical solution from the numerical solution is small. The final values we chose are $\omega_x, \omega_y \in [0.3, 3.2]$ and $c_x, c_y \in [0, 4.5]$, which gave an acceptable error of around 2.6% (defined according to the same loss metric used for network evaluation).

Results for using QHO potentials with analytically-defined solutions as the surrogate dataset is shown in Supplementary Figure 3d, where SIB-CL was seen to also outperform the baselines. However, we observe a smaller performance margin from the baselines, as well as between the two baselines, as compared to the other problems; this is likely due to the simplicity of the QHO surrogate dataset.

S4. ADDITIONAL BASELINES: INCLUDING INVARIANCE INFORMATION USING DATA AUGMENTATION

The baselines introduced in the main text, SL and TL, do not invoke any invariance information during network training. In Supplementary Figure 4, we explore adding invariance information to these baselines via a simple data augmentation approach; we refer to them as SL-I and TL-I for invariance-augmented SL and TL respectively. Specifically, the training procedure of SL-I corresponds exactly to Fig. 2c in the main text, i.e. each sample in the fine-tuning dataset undergoes a transformation randomly sampled from $\mathcal{G}$ before entering the encoder network $\mathbf{H}$. Similarly, TL-I corresponds exactly to Fig. 2bc, where

samples from the surrogate and target dataset are each transformed with a random element from $\mathcal{G}$ before entering the encoder $\mathbf{H}$ during both the pre-training and fine-tuning stages. In Supplementary Figure 4, we show results of these invariance-augmented baselines and compare them against SIB-CL, SL and TL for each of the four regression problems described in this work: DOS prediction (Supplementary Figure 4a) and band structure prediction (Supplementary Figure 4b) of 2D PhCs; and for TISE, we have ground-state energy prediction when using low resolution data (Supplementary Figure 4c) and QHO potentials (Supplementary Figure 4d) as surrogate datasets. In all four problems, we found that SIB-CL outperforms *all* the baselines, some with significant margins.

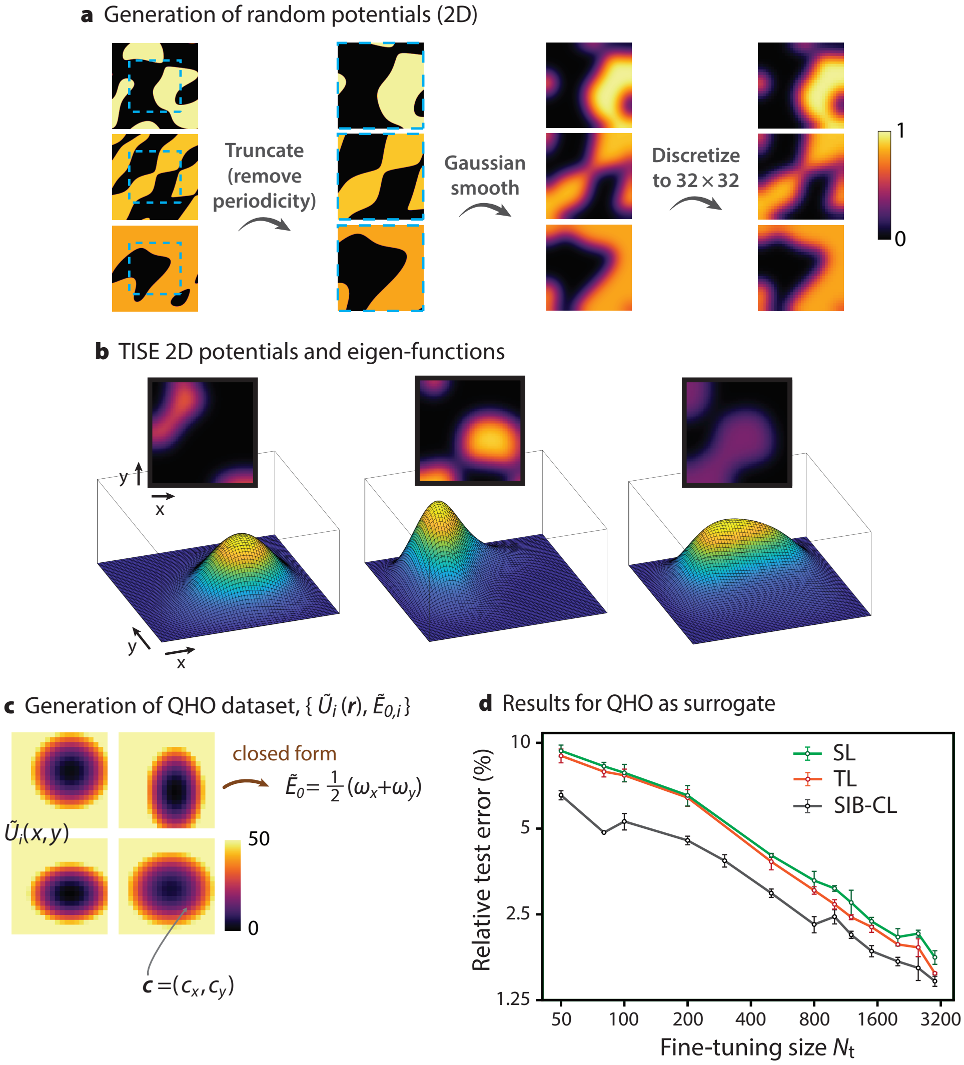

Supplementary Figure 3. TISE QHO a) Illustration of the proceedure used to generate samples of random potential, here illustrated in 2D for simplicity. **b**) Examples of 2D potentials and their corresponding wavefunction solutions (eigenfunctions) show that solutions are non-trivial. **c**) The QHO dataset consists of 2D QHO potentials, $\tilde{U}(\mathbf{r}) = \frac{1}{2}\omega^{\circ 2} \cdot (\mathbf{r}-\mathbf{c})^{\circ 2}$ with different values of ω and $\mathbf{c}$, and labels are the closed-form solutions of the QHO (ignoring Dirichlet boundary conditions). **d**) Results of SIB-CL (using BYOL for contrastive learning) compared against the baselines for ground-state energy prediction in 2D when using the QHO surrogate dataset. Error bars show the 1σ uncertainty level when varying the data selection of the fine-tuning dataset.

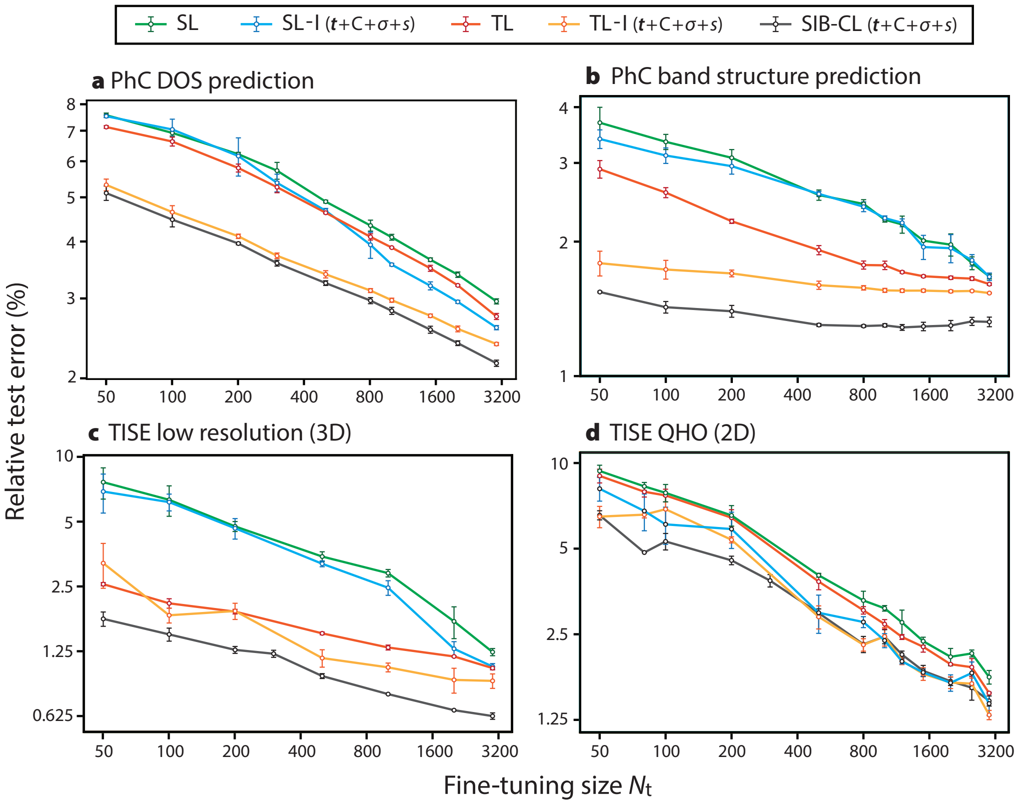

Supplementary Figure 4. Baselines with invariance information Results for supervised learning (SL), invariance-augmented supervised learning (SL-I), regular transfer learning (TL), invariance-augmented transfer learning (TL-I) and SIB-CL for all four regression problems discussed in this work: **a**) Density-of-states (DOS) prediction of 2D photonic crystals (PhCs), **b**) band structure prediction of 2D PhCs, **c**) ground-state energy prediction for 3D potentials using low resolution data as surrogate, and **d**) ground-state energy prediction for 2D potentials using quantum harmonic oscillator (QHO) potentials as surrogate. Error bars show the 1σ uncertainty level when varying the data selection of the fine-tuning dataset.

S5. "BAKING IN" INVARIANCES USING CONTRASTIVE LEARNING: ABLATION STUDIES AND FURTHER EXPERIMENTS

Using the notation in the main text, the group of invariant transformations used for the PhC DOS prediction problem can be expressed as $\mathcal{G} = \{1\,,\, \mathbf{t}\,,\, C\,,\, \sigma\,,\, s\}$, where we use $C = \{C_2, C_4^{\pm}\}$ and $\sigma = \{\sigma_h, \sigma_v, \sigma_d^{(\prime)}\}$ to indicate the set of all rotation and flip operations respectively. In Supplementary Figure 5a, we present results from an ablation experiment on SIB-CL, where we selectively, and increasingly, remove transformations from $\mathcal{G}$. We observe SIB-CL's prediction accuracy to *monotonically worsen (improve)* as more transformations are removed (added) from $\mathcal{G}$. This is expected, since all of these transformations are *true* invariances of the (DOS prediction) problem, and thus including more types of transformation is equivalent to incorporating more prior knowledge of the problem which should improve the model accuracy. This observation is typically not echoed in standard contrastive learning applications in computer vision; a possible reason is that the data augmentation strategies used to generate the transformations there are not necessarily *true* invariances since the downstream task is not known during contrastive learning and thus some transformations may prove ineffective [S5]. This observation also validates the effectiveness of using contrastive learning to invoke prior knowledge of physical invariances.

Next, we also performed an ablation experiment where we discarded all invariance information from the contrastive learning step, for both SimCLR and BYOL. To do so, we reduce $\mathcal{G}$ to the trivial identity group, $\mathcal{G} = \{1\}$, (i.e. such that $g\mathbf{x}_i = g'\mathbf{x}_i = \mathbf{x}_i$ in Fig. 2 of the main text). The results of this experiment are shown in Supplementary Figure 5b, where we observe that both SimCLR and BYOL gave prediction accuracies

that coincided very well to the TL baseline (which includes only information from the surrogate dataset and not invariances). This further validates SIB-CL's approach of using contrastive learning to invoke knowledge of invariances—once this knowledge is removed, doing contrastive learning as a pre-training step provides *no benefits*. Equivalently, this suggests that the utility of SIB-CL is derived from attracting non-trivial positive pairs (i.e. learning invariances) rather than repelling negative pairs.

Another insightful ablation experiment is to retain all elements of $\mathcal{G}$ during pre-training, but reduce $\mathcal{G}$ to the trivial identity group only during fine-tuning. This experiment can be explored for SIB-CL and TL-I (but not SL-I since doing so would give exactly SL) and will tell us how effectively each method had "baked in" invariance information into the represention during the pre-training stage. We refer to these experiments as SIB-CL-rt and TL-I-rt correspondingly. From Supplementary Figure 5c, the high correspondence observed between SIB-CL and SIB-CL-rt suggests that SIB-CL learns, and retains, most of the invariance information from the pre-training stage. On the contrary, the gap between TL-I and TL-I-rt suggests that while some invariance information is learnt during pre-training (indicated by the gap between TL and TL-I-rt), much of its "invariance learning" also occurs during the fine-tuning stage. This suggests that, compared to TL-I, representions learnt from SIB-CL have higher semantic quality; this is highly desirable, for example, in domains where there exist a series of different tasks (that obey the same invariances) since these representations can be simply "reused" without needing to re-train the network.

We also studied three different algorithms for performing contrastive learning on these invariances, which are described in detail as follows;

1. Standard: transformations from $\mathcal{G}$ are uniformly sampled from the sub-groups in the following order: $[\mathcal{G}_{\mathbf{t}}, \mathcal{G}_0, \mathcal{G}_{\mathrm{s}}]$ and applied sequentially to each input. This sampling procedure gives rise to instances which have "highly compounded" invariances; in other words, the two variations of the unit cell (or a positive pair) can look drastically different.

2. Independent: contrastive learning was performed independently for each sub-group of transformations and their losses were summed; i.e. we create a positive pair for *each* sub-group, compute the NT-Xent loss for each pair and sum them to get the total contrastive loss. This algorithm is slower than the rest (though it can be mitigated via parallel computing) since the number of forward passes per iteration increases according to the number of sub-groups present. However, this sampling procedure create positive instances that differ only by a single sub-group operation and hence their differences are less drastic.

3. Stochastic: Similar to standard, except there is some probability, p_α, to whether each transformation sampled from $\mathcal{G}_\alpha$ will be applied. In our experiments, we set $p_\alpha = 0.5$ for all α's, (i.e. $\alpha \in \{0, \mathbf{t}, \mathrm{s}\}$). In other words, there is a 50% chance we do not apply each transformation.

These 3 algorithms are compared in Supplementary Figure 5d, where the stochastic approach is seen to produce the best model accuracies. While the differences are small, the stochastic method is still strictly better than the other two for various combinations of transformations (Supplementary Figure 5d left & right) and statistically significant at larger N_t. We conjecture that this is because the standard method uses instances with highly compounded invariances which are difficult to learn, whereas the independent method uses simple invariances governed by single transformations which are easily learnt but omits useful knowledge of compounded invariances. The stochastic method presents as an ideal middle-ground between the two methods. In practice, the individual p_α for each sub-group of transformations are hyper-parameters that should be tuned for optimization; a detailed tuning of the individual p_α will likely widen the performance edge of the stochastic method.

Finally, further ablation experiments were conducted to better understand the contributions from each of the individual components in isolation. In particular, we compare SIB-CL against the most standard approaches of pre-training: transfer learning and contrastive learning (Supplementary Figure 5e). For transfer learning, the network was first pre-trained on the surrogate dataset and transfered to the target problem (without invariances) and for contrastive learning, we applied the standard approach of unsupervised pre-training by contrasting different invariance-related variations. Supplementary Figure 5e shows

Problem	Pre-training	Fine-tuning
DOS	$B \in \{16, 32, 64, 128\}$<br>$\alpha \in \{10^{-5}, 10^{-4}, 10^{-3}\}$	$B \in \{16, 32, 64, 128\}$<br>$\alpha \in \{10^{-4}, 5\cdot10^{-4}, 10^{-3}, 5\cdot10^{-3}\}$
Bandstructure	$B \in \{16, 32, 64\}$<br>$\alpha \in \{10^{-4}, 10^{-3}\}$	$B \in \{16, 32, 64\}$<br>$\alpha \in \{10^{-4}, 10^{-3}\}$
TISE	$B \in \{32, 64, 128\}$<br>$\alpha \in \{10^{-5}, 10^{-4}\}$	$B \in \{32, 64, 128\}$<br>$\alpha \in \{10^{-4}, 10^{-3}\}$

Supplementary Table 3. Set of hyperparameters the baseline methods–transfer learning (TL), transfer learning with invariances augmentation (TL-I), supervised learning (SL) and supervised learning with invariance augmentations (SL-I)–were tuned over. For SL and SL-I, there is no pre-training and only the set of hyperparameters listed under fine-tuning were used. The main hyperparameters varied in all experiments are the batch size (B) and the learning rates (α).

that both the constrastive learning and transfer learning stages contribute to the performance of SIB-CL, with contrastive learning being the dominant contributor.

S6. ADDITIONAL COMMENTS ON HYPERPARAMETERS

To ensure a fair evaluation of SIB-CL against the baselines, we allocate a similar level of effort to tune the hyperparameters of the baseline methods. In Supplementary Table 3, we show the set of hyperparameters that the baseline methods were tuned over.

An important hyperparameter in our experiments is the amount of pre-training; as described in the main text, we saved the pre-trained model at $\{100, 200, 400\}$ epochs for SIB-CL, and at $\{40, 100, 200\}$ epochs and for TL baselines (both with and without invariances). Here, we expand upon our "seemingly-arbitrary" choice of checkpoints. Ideally, the model should be fine-tuned after every epoch of pre-training to find the most optimal initialization; this would however be computationally unrealistic since the fine-tuning stage also has an extensive set of hyperparameters to optimize over. As such, we only select the model initializations at discrete intervals. We used different checkpoints for the TL baselines (as opposed to SIB-CL) because we observed that the TL methods were prone to overfit while SIB-CL was not (we hypothesise that this is because the contrastive loss objective provides implicit regularization). This is illustrated in Supplementary Figure 6, where we observe that the test loss for the TL methods starts to increase beyond $\approx 20 - 100$ epochs of pre-training and hence do not select checkpoints beyond 200 pre-training epochs. From Supplementary Figure 6, we further note that while we capped the pre-training duration to 400 epochs in order to reduce computational requirements, SIB-CL's test lost was observed to decrease beyond 400 epochs and thus we can potentially attain higher data efficiencies than the ones reported in this paper if we perform longer pre-training.

Apart from the hyperparameters discussed in the Methods section of the main text, SIB-CL involves many other possible training hyperparameters which was not studied in this work. For instance, we simply alternate contrastive learning and predictor pre-training (i.e., Fig. 2ab in the main text) after *every* epoch; however, one could in principle vary the interval between the two steps, perform the two steps entirely sequentially, or even consider joint-training (i.e. updating the weights of $\mathbf{H}$ using a weighted average of the loss from both steps). The initial motivation for training the two steps alternately (instead of sequentially) is to provide some information about the predictive task to $\mathbf{H}$ during contrastive learning. This is a key deviation of SIB-CL from standard self-supervised learning (SSL) techniques which are completely task agnostic. We conjecture that SIB-CL will be more effective for predictive modelling problems in science, where the labels are often more sophisticated (and the label space is often larger) than image classes used in vision tasks for standard SSL. For standard SSL, a single linear layer (as opposed to a dense non-linear predictor network used in our case) is often applied after the encoder and is deemed to be sufficient for the classification task; this is a common evaluation procedure in SSL [S3; S4; S9] also better known as the "linear protocol".

Additionally, as suggested in Section S5, the stochastic sampling parameter for the different sub-group

Method	Time to build/initialize model (s)	Time for single forward pass(s)
SIB-CL	0.046	0.0098
E2CNN	56.5	1.33

Supplementary Table 4. Computational times for building the network and performing a single forward pass of the network in SIB-CL vs in E2CNN models of equal number of parameters (approx. 8M parameters).

of invariances (i.e. p_α) can also be tuned separately (here, we set them all to 0.5 for simplicity). Tuning the frequency and strength of transformations was found to be highly important in contrastive learning in standard computer vision applications [S5; S7] and are thus often exhaustively varied during optimization [S1]. If we were to study the variations discussed here, performances better than the ones presented in this work might be attainable for SIB-CL.

S7. COMPARISON WITH STATE-OF-THE-ART EQUIVARIANT NETWORK

Given that the concept of SIB-CL is highly complementary to the large body of work on symmetry-preserving architectures, it is instructive to contrast SIB-CL against such methods. Here, we show results when comparing SIB-CL against a state-of-the-art equivariant network, the E(2)-Equivariant CNNs proposed by Weiler and Cesa [S6] which we denote E2CNN here. We reproduced the code from the Github repository* provided by the authors and for a fair comparison between E2CNN and SIB-CL, we scale the width of E2CNN such that the number of parameters are approximately equal to the number of parameters in SIB-CL† (the authors also use a similar technique to ensure a fair comparison of their model with their Wide-ResNet [S8] baseline).

The results are shown in Supplementary Figure 7, where we used the default configuration of E2CNN. Since this default configuration does not account for periodic translation symmetry, we compared it against SIB-CL when using just rotations and mirrors as invariances for a fair comparison. Experiments were conducted for E2CNN trained with (E2CNN-TL) and without (E2CNN-SL) an additional pre-training stage on using the surrogate data. For E2CNN-TL we follow the training proceedure of the non-equivariant TL baseline. SIB-CL was observed to perform comparably to E2CNN-TL and even performing slightly better at larger dataset sizes, despite its simplicity in implementation (without the use of hand-tailored symmetry-respecting kernels). More strikingly, we observed that SIB-CL was computationally much faster to train compared to E2CNN, with the computational inefficiencies in E2CNN likely arising from intricate calculations needed to enforce the symmetry-preserving kernel operations. As shown in Supplementary Table 4, each forward pass of the E2CNN model is more than 100× slower than SIB-CL, potentially presenting a severe bottleneck not just for training the network, but also during inference. In contrast, SIB-CL's fast computation times is highly desirable especially if the primary motivation of using deep learning is to have a trained model that can perform inference much faster than the original numerical method.

In addition, we also note that equivariant networks like E2CNN are often limited to geometric symmetries of the input since equivariance is guaranteed by enforcing them in every operation of sequential layers in the network. On the other hand, SIB-CL is able to enforce a wider range of invariances, including non-symmetry related or physics-informed invariances of the problem, due to its simple approach of enforcing them via contrasting random samples. An example of such invariances that was presented in this work was the use of refractive scaling in the DOS prediction problem.

---

* https://github.com/QUVA-Lab/e2cnn

† the total number of parameters in the encoder and predictor networks of SIB-CL used in the DOS prediction problem is approximately 8M

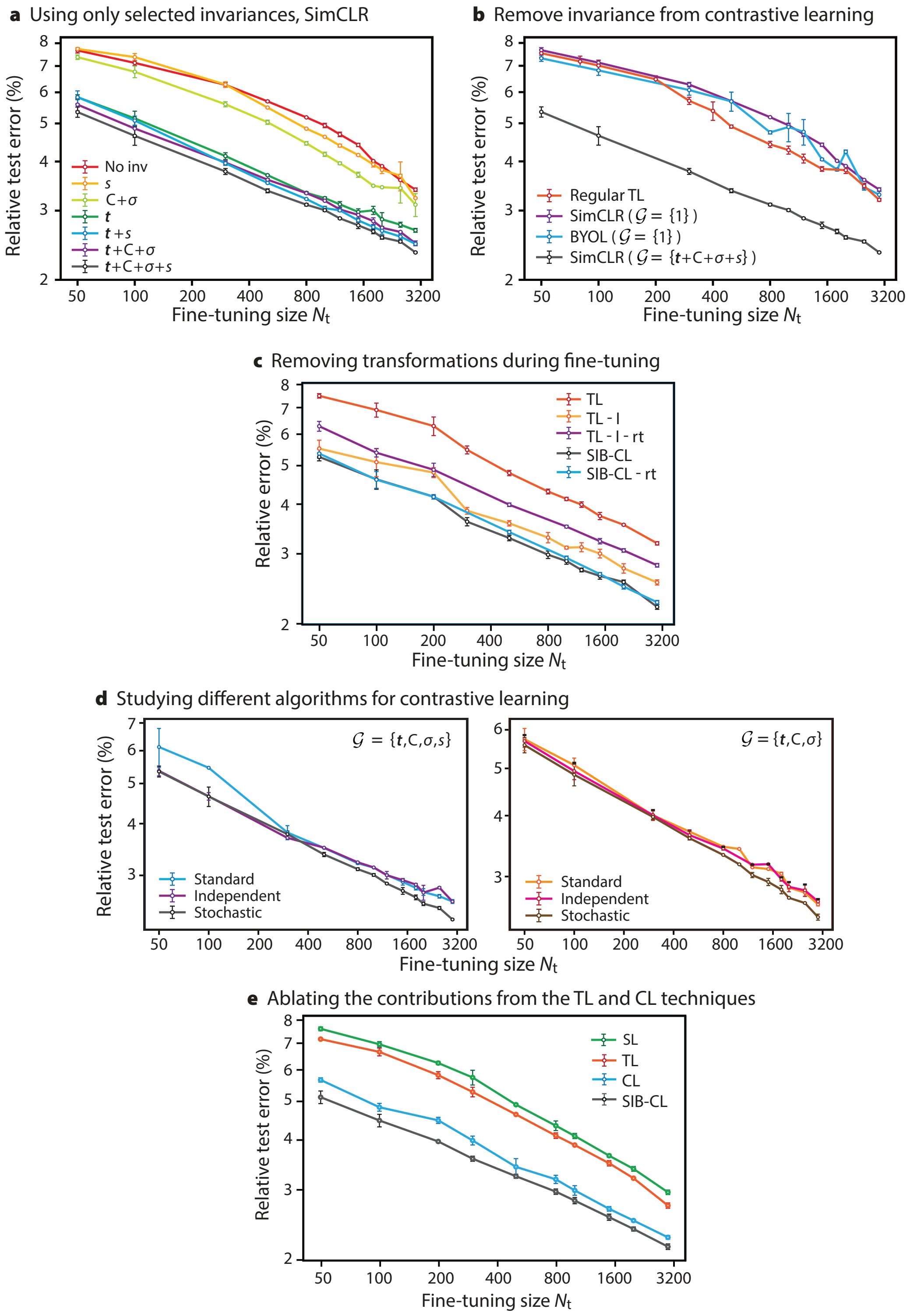

Supplementary Figure 5. Ablation studies Results from ablation experiments on the DOS prediction task show that: **a**) when selected transformations are removed from the group of known invariances $\mathcal{G}$ during contrastive learning, performance of SIB-CL (here based on SimCLR) monotonically worsens; **b**) when reducing $\mathcal{G}$ to just the trivial identity group (during both pre-training and fine-tuning), both SimCLR and BYOL display similar performance to the transfer learning baseline (without invariances); **c**) when reducing $\mathcal{G}$ to just the trivial identity group only during fine-tuning (here referred to by appending -rt to the methods' abbreviations), SIB-CL's performance was not compromised while TL-I's was; **d**) the stochastic sampling approach (explained in the text) produces the best performance, both when all transformations are included (left) and all but scaling transformation are included (right). **e**) Isolating the contributions from each of the techniques used in SIB-CL, TL refers to regular transfer learning utilizing the surrogate dataset (without invariances) and CL refers to using pure contrastive learning (without the surrogate dataset) during pre-training on the same architecture. Error bars show the 1σ uncertainty level when varying the data selection of the fine-tuning dataset.

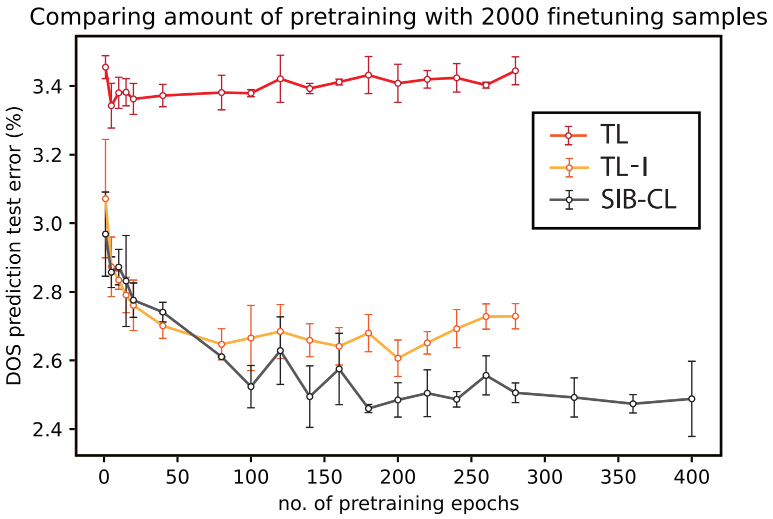

Supplementary Figure 6. Prediction error against number of pre-training epochs for SIB-CL vs the TL baselines on the DOS prediction problem. The model was saved at finer intervals of pre-training and each saved model was fine-tuned with 2000 labeled examples to derive this plot. The plots show the minimum test loss (averaged over 3 seeds, 1σ level indicated by the error bars) achieved within the full space of pre-training and fine-tuning hyperparameters.

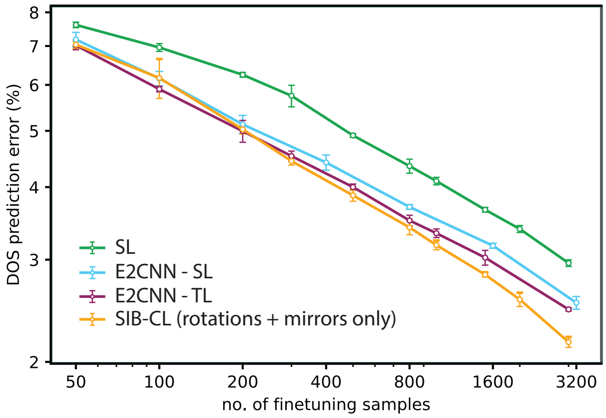

Supplementary Figure 7. Comparison of SIB-CL with an equivariant network E2CNN [S6]. E2CNN is trained using supervised learning (E2CNN-SL) as well as fine-tuned after an additional pre-training stage using the surrogate dataset (E2CNN-TL). The supervised baseline (SL) using the non-equivariant architecture that SIB-CL uses is also included for comparison. Error bars show the 1σ uncertainty level when varying the data selection of the fine-tuning dataset.

SUPPLEMENTAL REFERENCES

[S1] Chen, Ting, Simon Kornblith, Mohammad Norouzi, and Geoffrey Hinton (2020), "A Simple Framework for Contrastive Learning of Visual Representations," arXiv:2002.05709 .

[S2] Grill, Jean-Bastien, Florian Strub, Florent Altché, Corentin Tallec, Pierre H. Richemond, Elena Buchatskaya, Carl Doersch, Bernardo Avila Pires, Zhaohan Daniel Guo, Mohammad Gheshlaghi Azar, Bilal Piot, Koray Kavukcuoglu, Rémi Munos, and Michal Valko (2020), "Bootstrap your own latent: A new approach to self-supervised Learning," arXiv:2006.07733 .

[S3] Kolesnikov, Alexander, Xiaohua Zhai, and Lucas Beyer (2019), "Revisiting Self-Supervised Visual Representation Learning," arXiv:1901.09005 .

[S4] Oord, Aaron van den, Yazhe Li, and Oriol Vinyals (2019), "Representation Learning with Contrastive Predictive Coding," arXiv:1807.03748 .

[S5] Tian, Yonglong, Chen Sun, Ben Poole, Dilip Krishnan, Cordelia Schmid, and Phillip Isola (2020), "What Makes for Good Views for Contrastive Learning?" arXiv:2005.10243 .

[S6] Weiler, Maurice, and Gabriele Cesa (2019), "General $E(2)$-Equivariant Steerable CNNs," arXiv:1911.08251 .

[S7] Xiao, Tete, Xiaolong Wang, Alexei A. Efros, and Trevor Darrell (2021), "What Should Not Be Contrastive in Contrastive Learning," arXiv:2008.05659 .

[S8] Zagoruyko, Sergey, and Nikos Komodakis (2016), "Wide residual networks," CoRR **abs/1605.07146**, 1605.07146.

[S9] Zhang, Richard, Phillip Isola, and Alexei A. Efros (2016), "Colorful Image Colorization," arXiv:1603.08511 .
